# Rapid Identification of Novel Immunodominant Proteins and Characterization of a Specific Linear Epitope of *Campylobacter jejuni*

**DOI:** 10.1371/journal.pone.0065837

**Published:** 2013-05-29

**Authors:** Sebastian Hoppe, Frank F. Bier, Markus v. Nickisch-Rosenegk

**Affiliations:** 1 Fraunhofer Institute for Biomedical Engineering, Am Muehlenberg, Potsdam, Germany; 2 University Potsdam, Institute of Biochemistry and Biology, Potsdam, Germany; Federal University of São Paulo, Brazil

## Abstract

*Campylobacter jejuni* remains one of the major gut pathogens of our time. Its zoonotic nature and wide-spread distribution in industrialized countries calls for a quick and reliable diagnostic tool. Antibody-based detection presents a suitable means to identify pathogenic bacteria. However, the knowledge about immunodominant targets is limited. Thus, an approach is presented, which allows for the rapid screening of numerous cDNA derived expression clones to identify novel antigens. The deeper understanding of immunodominant proteins assists in the design of diagnostic tools and furthers the insight into the bacterium’s pathogenicity as well as revealing potential candidates for vaccination. We have successfully screened 1536 clones of an expression library to identify 22 proteins that have not been described as immunodominant before. After subcloning the corresponding 22 genes and expression of full-length proteins, we investigated the immunodominant character by microarrays and ELISA. Subsequently, seven proteins were selected for epitope mapping. For cj0669 and cj0920c linear epitopes were identified. For cj0669, specificity assays revealed a specific linear epitope site. Consequently, an eleven amino acid residue sequence TLIKELKRLGI was analyzed via alanine scan, which revealed the glycine residue to be significant for binding of the antibody. The innovative approach presented herein of generating cDNAs of prokaryotes in combination with a microarray platform rendering time-consuming purification steps obsolete has helped to illuminate novel immunodominant proteins of *C.jejuni*. The findings of a specific linear epitope pave the way for a plethora of future research and the potential use in diagnostic applications such as serological screenings. Moreover, the current approach is easily adaptable to other highly relevant bacteria making it a formidable tool for the future discovery of antigens and potential biomarkers. Consequently, it is desirable to simplify the identification of structural epitopes, as this would extend the spectrum of novel epitopes to be detected.

## Introduction

*C. jejuni* is a Gram-negative, microaerophilic bacterium possessing a helical-shaped morphology [1]. In industrialized countries, *C. jejuni* has been one of the primary causal agents of gastroenteritis. In 2012, in Germany alone 62626 cases have been reported [2]. Campylobacteriosis predominantly induces mild, self-limiting diarrhoea, however severe cases have been reported [3]. Several studies have shown the potential contribution of Campylobacteriosis in the development of neuropathies such as the Guillain-Barré syndrome [4], [5]. The prominent route of infection is the improper handling and insufficient cooking of poultry [6]. The broad distribution of this pathogen in combination with a high clinical relevance necessitates fast and reliable diagnosis. Although several genomic typing methods exist [7], [8], these are often time-consuming and inappropriate for a point-of-care application. Instead, a direct approach detecting the whole bacterium is beneficial. This can be achieved by using specific antibodies to membrane-associated antigens similar to the Latex-Agglutination-Test that is already available for several bacterial pathogens including *Campylobacter* [9].

In order to achieve this, copious knowledge of potentially suitable targets, i.e. immunodominant proteins, is indispensable. In the past, screening for immunogenic proteins has been carried out on nitrocellulose membranes [10] or using microarray library screening with extensive protein purification [11]. However, these methods have some major drawbacks as the former is prone to non-specific binding and cross-reactivity when using polyclonal sera for screening of bacterial libraries [12], while the latter method is time-consuming and laborious due to the purification steps needed prior to microarray printing.

As we have shown elsewhere, an approach using HaloTag® and specifically coated HaloLink™ slides is better suited to detect immunodominant proteins while reducing cross-reactivity to a minimum [13]. The HaloTag® provides several advantages to other commonly used tags as it enhances the amount of soluble proteins expressed [14] reducing the formation of inclusion bodies. In addition, the interaction of tag and its specific ligand is based on covalent binding [15]. This negates the need for additional purification steps as the crude lysate can simply be spotted onto coated microarrays. Only the target proteins presented as fusion constructs bearing a HaloTag® will bind to the surface, whereas the remaining proteins are washed off.

Combining the described screening method with expression libraries derived from *C. jejuni* cDNA allows for the fast analysis of hundreds of different proteins. Thus, suitable immunodominant proteins can be detected, isolated and identified via sequencing the encoding cDNA sequence.

The generation of a cDNA derived expression library offers advantages in contrast to genomic libraries. The latter demand excessive screenings as the genetic information is mostly truncated or of little relevance representing areas within the genome that do not encode for proteins, whereas the former focuses on the genes transcribed [16]. This reduces the amount of clones to be screened.

Nevertheless, for effective cDNA library screening normalization is needed, as rRNA is mainly overrepresented due to its extreme abundance within a total RNA extraction prior to reverse transcription [17]. Bacteria only posses a poly(A)-tail on their mRNA in rare cases [18], [19]. Although methods exist to isolate mRNA from bacteria [20], [21], it is generally considered to be more challenging as compared to eukaryotic RNA, where Oligo(dT) primers are sufficient [22]. Therefore, we refrained from isolating the mRNA prior to reverse transcription. Instead, the generated cDNA was normalized, i.e. trimmed down, afterwards by the use of a duplex-specific nuclease [23]. This approach has been shown to effectively reduce the amount of rRNA-derived molecules, thereby altering the overall composition in favour of the mRNA-derived cDNA without including a bias [24]. Further optimization of library construction was achieved by using a ligation-independent cloning as well as electroporation, which have been shown to enhance overall cloning efficiency [25], [26].

Using this approach, a relatively small number of clones can be screened to illuminate immunodominant proteins effectively. The identification of previously unknown or incompletely described antigens offers several benefits and potential applications. First, these proteins might serve in a diagnostic tool to rapidly identify *C. jejuni* in biological samples, e.g. in food manufacturing industry or hospitals. Secondly, proteins eliciting an immune response might be suitable candidates for vaccination. Finally, gaining insight into the structure and function of novel immunodominant proteins might improve the overall understanding of a bacterium’s pathogenicity as it could accelerate the elucidation of novel virulence-associated factors.

In this paper, we show the successful screening of an expression library of *C. jejuni* identifying several potentially immunodominant proteins. After further investigations, we selected a subset of these proteins for epitope mapping and succeeded in identifying linear epitopes for two proteins, namely cj0669 and cj0920c not described before. Furthermore, assays were performed to assess specificity of the binding as well as investigating the relevance of the amino acid residues involved via alanine scanning. Additionally, the structure and antigenicity of the proteins and epitopes were modelled to analyze the suitability of the identified sequences for future applications like diagnostic tools or vaccine development.

## Results

### Library creation and normalization

The RIN values for the *C. jejuni* RNAs isolated were above 8.5 in all cases (see S1: RIN). After polyadenylation of the RNA, it was reverse transcribed and subsequently normalized using a duplex-specific nuclease (DSN). Assessing the performance of normalization, we sequenced a sample set of 96 individual clones after transformation with trimming and without trimming. Without DSN treatment 28% of clones contained 23S rRNA derived cDNA and 8% other rRNA derived cDNA (16S and 5S). In comparison, after incubation with DSN only one clone in 96 showed a 23S rRNA derived cDNA.

### Screening of cDNA expression library

A total number of 1536 different clones were screened using HaloLink™ slides. We used HisJ (UniProt/Swiss-Prot: Q46125), CjaA (Q0P9S0) and Peb1a (Q0P9X8) as positive reference markers, as they have been described previously to be immunodominant [27]–[29]. In contrast, ArgC (Q9PIS0) and PyrC (Q0PBP6) were used as negative reference proteins. After comparing the median value and the standard deviation of each sample to the values of the positive references, a selection of 192 clones were picked to be sequenced. Generally, we grouped clones into three categories after screening. Group I included samples showing a higher median contrast value than all the positive references used, group II encompassed the samples with median contrast values in between those of different positive references and group III the remaining samples which albeit below the lowest positive reference were still above all the negative reference signals. After screening 1536 clones, 32% fell into group I, 15% encompassed group II and 4% made up group III while the remaining 50% were below the negative controls. The sequenced clones were “blasted” against the genome of *C. jejuni* NCTC 11168 (RefSeq: NC_002163) to identify the corresponding genes and proteins. For further experiments, known antigens of *C. jejuni* found within the sequenced clones were discarded and we focused solely on 22 novel potentially immunogenic proteins. Several of the identified clones carried only truncated fragments of the corresponding genes. In addition, some of the sequenced inserts possessed a frame shift. In order to overcome these limitations full-length inserts were prepared and subcloned to express the full-length proteins.

### Characterization and analysis of immunodominant behaviour

**Figure 1:**
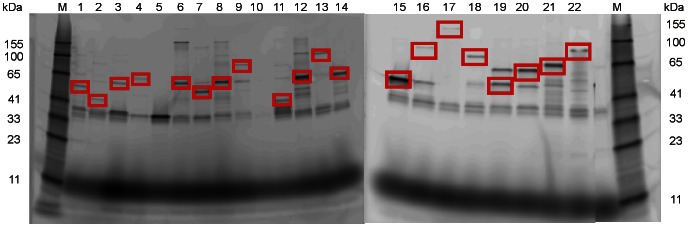
SDS-PAGE of recombinantly expressed fusion constructs. The whole lysates labelled by incubation with HaloTag® Alexa 488 ligand were separated by SDS-PAGE. M refers to the BenchMark™Fluorescent Protein Standard (Invitrogen). For almost all proteins, bands were visible with the respective sizes of their molecular weight and the HaloTag™. The HaloTag™ alone at 34 kDa is visible throughout. The proteins were as follows: 1 – cj0476, 2 – cj1723, 3 – cj1208, 4 – cj1619, 5 – cj0920c, 6 – cj1380, 7 – cj1381, 8 – cj0669, 9 – cj1624, 10 – cj1320, 11 – cj1486, 12 – cj0130, 13 – cj1366, 14 – cj0016, 15 – cj1575, 16 – 1576, 17 – cj1729, 18 – cj0571, 19 – cj0926, 20 – cj0927, 21 – cj0623 and 22 – cj1138. The pictures of the two gels as well as the righthand marker were fused by CorelDraw to minimize space. For all proteins except cj0920c (5) and cj1320 (10) bands are visible. In lane 4, cj1619 shows a band at around 65 kDa whereas the fusion protein ought to show a size of approximately 80 kDa. The correct-sized fusion proteins are highlighted in red.

The 22 genes and their corresponding proteins are summarized in Table 1 including the length as well as the size of the protein without the attached HaloTag®. Recombinant expression of the full-length fusion proteins was assessed by SDS-PAGE using a HaloTag® 488 Alexa ligand, see Figure 1. The correct-sized proteins are highlighted as they are expressed fused to the 34 kDa HaloTag®. Bands at roughly 34 kDa in size are visible throughout, likely representing early-termination transcripts comprised only of the HaloTag®.

**Table 1:** The table lists 22 novel immunodominant proteins.

Table 1. Immunodominant protein candidates Locus Tag [UniProt/Swiss-Prot]	Protein	length [aa]	Size [kDa]	Mean Q ± s.d. [n = 25]
Cj0016 [P0C634]	7-cyano-7-deazaguanine synthase	224	25.1	0.64±0.18
Cj0130 [Q0PC07]	prephenate dehydrogenase	275	30.9	0.70±0.19
Cj0476 [Q9PI33]	50S ribosomal Protein L10	159	17.7	0.40±0.13
Cj0571 [Q0PAU6]	Putative transcriptional regulator	290	34.3	0.52±0.21
Cj0623 [Q0PAP4]	Hydrogenase isoenzymes formation protein	247	27.5	1.33±0.47
Cj0669 [Q0PAK5]	ABC-transporter ATP-binding protein	242	26.6	0.59±0.21
Cj0920c [Q0P9X9]	Putative ABC-type amino-acid transporter permease	250	28	0.49±0.18
Cj0926 [Q0P9X3]	Putative membrane protein	110	13	1.77±0.75
Cj0927 [Q9PP06]	Adenine phosphoribosyltransferase	182	20.6	2.23±0.58
Cj1138 [Q7BC53]	Putative galactosyltransferase	389	46.4	1.16±0.57
Cj1208 [Q0P948]	5-formyltetrahydrofolate cyclo-ligase	208	25.2	0.71±0.20
Cj1320 [Q0P8T7]	Putative aminotransferase	384	43.4	0.40±0.17
Cj1366c [Q9PMT4]	glucosamine-fructose-6-phosphate aminotransferase	598	67.1	0.69±0.23
Cj1380 [Q0P8M9]	Putative periplasmic protein	236	26.5	1.57±0.39
Cj1381 [Q0P8M8]	lipoprotein	176	20.4	0.38±0.12
Cj1486c [Q0P8D1]	Hypothetical protein	73	8.1	0.20±0.27
Cj1575c [Q0P853]	NADH-quinone oxidoreductase subunit E	75	8.7	1.21±0.46
Cj1576c [Q9PM99]	NADH-quinone oxidoreductase subunit D	408	46.9	1.55±0.43
Cj1619 [Q0P809]	alpha-ketoglutarate permease	419	46	0.32±0.12
Cj1624c [Q0P804]	L-serine dehydratase	454	49.4	0.61±0.30
Cj1723c [Q0P7Q8]	Putative periplasmic protein	74	7.8	0.34±0.14
Cj1729c [Q0P7Q2]	Flagellar hook subunit protein	865	91.9	0.59±0.20

The mean Q value (n = 25) was calculated and represents the respective signal intensity normalized to the median intensities of known antigens, HisJ and CjaA. The associated error was determined using error propagation by Gauss.

Evaluating immunodominance of these proteins, we performed 32 microarray and ELISA analyses using three different primary antibodies against *C. jejuni*. Summing up these results, Table 1 shows the respective mean Q values and errors (n = 25) for each of the proteins identified. The highest scores were attained by cj0926 and cj0927 with 1.77±0.75 and 2.23±0.58 respectively, indicating a potentially stronger immunogenicity as compared to the known immunodominant proteins HisJ and CjaA. However, we restrained from further characterizing the former two proteins via epitope mapping as both are highly conserved and show multiple homologies in cellular organisms (cj0927) or epsilonproteobacteria (cj0926) according to the NCBI protein cluster database [30]. The same rationale was applied for cj1576, which is conserved in cellular organisms and peaks at 1.55±0.43. Instead, we focused on cj0623, cj1575 and cj0669, which are highly conserved in *Campylobacter* only as well as cj0920c and cj1380, which in turn are conserved in Campylobacteraceae. Cj1380, cj0623 and cj1575c represent proteins with high mean Q values of 1.57±0.39, 1.33±0.47 and 1.21±0.46 respectively. Furthermore, we chose two proteins from an intermediate mean Q value range, cj0669 and cj0920c with 0.59±0.21 and 0.49±0.18. Both proteins belong to the protein family of ATP-binding cassette (ABC) transporters, which are partially situated on the outside of the bacterium [31]–[34] and thus of utmost importance in the context of this research. Finally, two proteins showing relatively low mean Q values of 0.40±0.13 and 0.34±0.14 for cj0476 and cj1723 were picked to complete the selection of proteins for epitope mapping.

### Epitope Mapping

The overlapping peptide sequences corresponding to each protein were mapped with three different primary antibodies to *C. jejuni*. Each peptide contains 15 amino acid residues with an overlap of 11 to each adjacent peptide.

**Figure 2:**
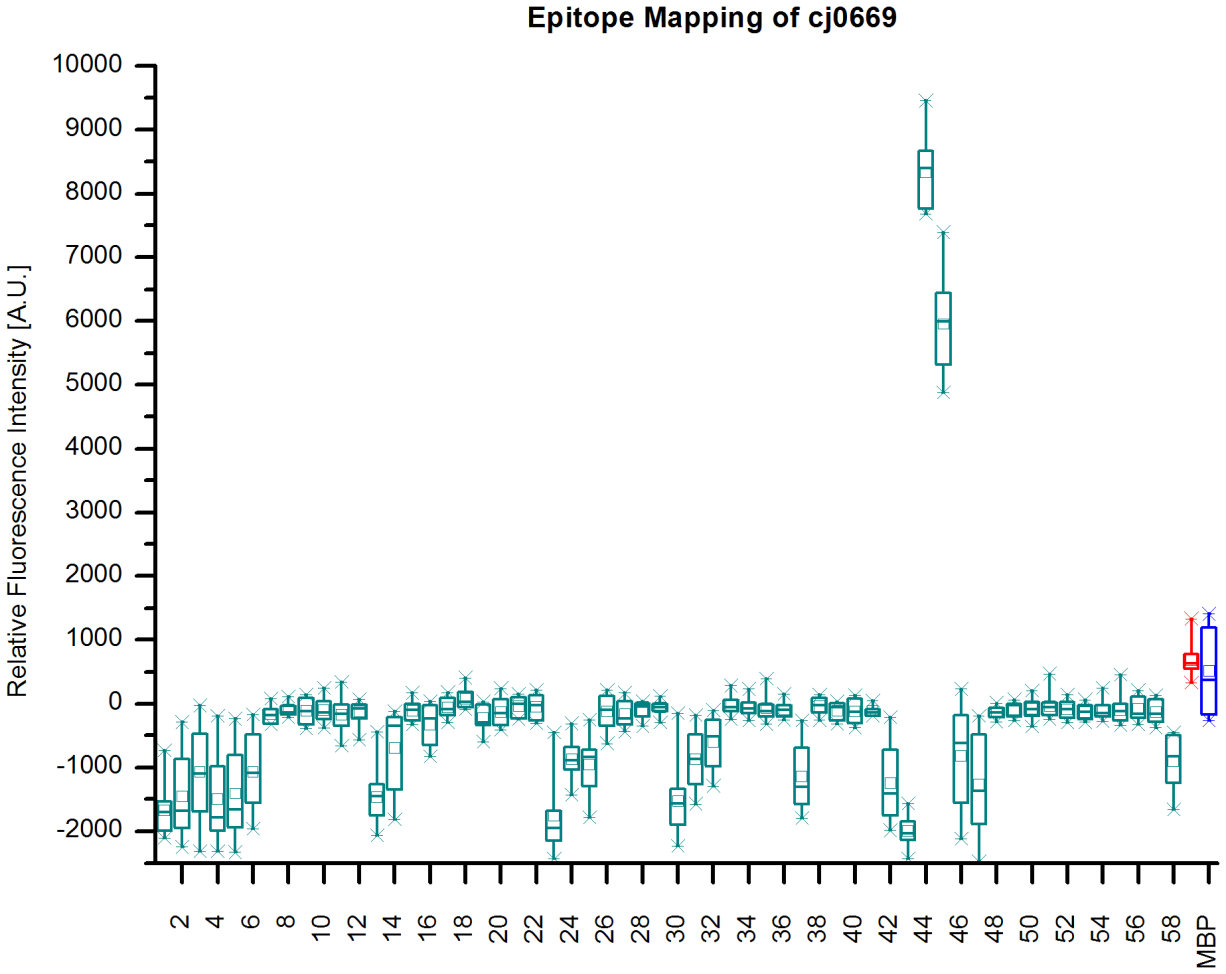
Epitope Mapping of cj0669. The Box-whisker-plot (n = 12) shows relative fluorescence intensities of the overlapping 58 peptides comprising cj0669. Rabbit Immunoglobulin G (red box) and Myelin Basal Protein (MBP, blue) are included as positive and negative controls respectively. Each box represents 50% of the values with the central horizontal line indicating the median value. The mean value for each sample is represented by a small rectangle. The whiskers include 98% of the data. Peptides 44 and 45 show significantly higher intensities with mean values above 8000 A.U. for peptide 44 and around 6000 A.U. for peptide 45 compared to approximately 1000 A.U. for Rabbit IgG indicating a strong interaction of the antibody to these peptides. Incubation was performed with three polyclonal antibodies reactive to C. jejuni and the results combined.

**Figure 3:**
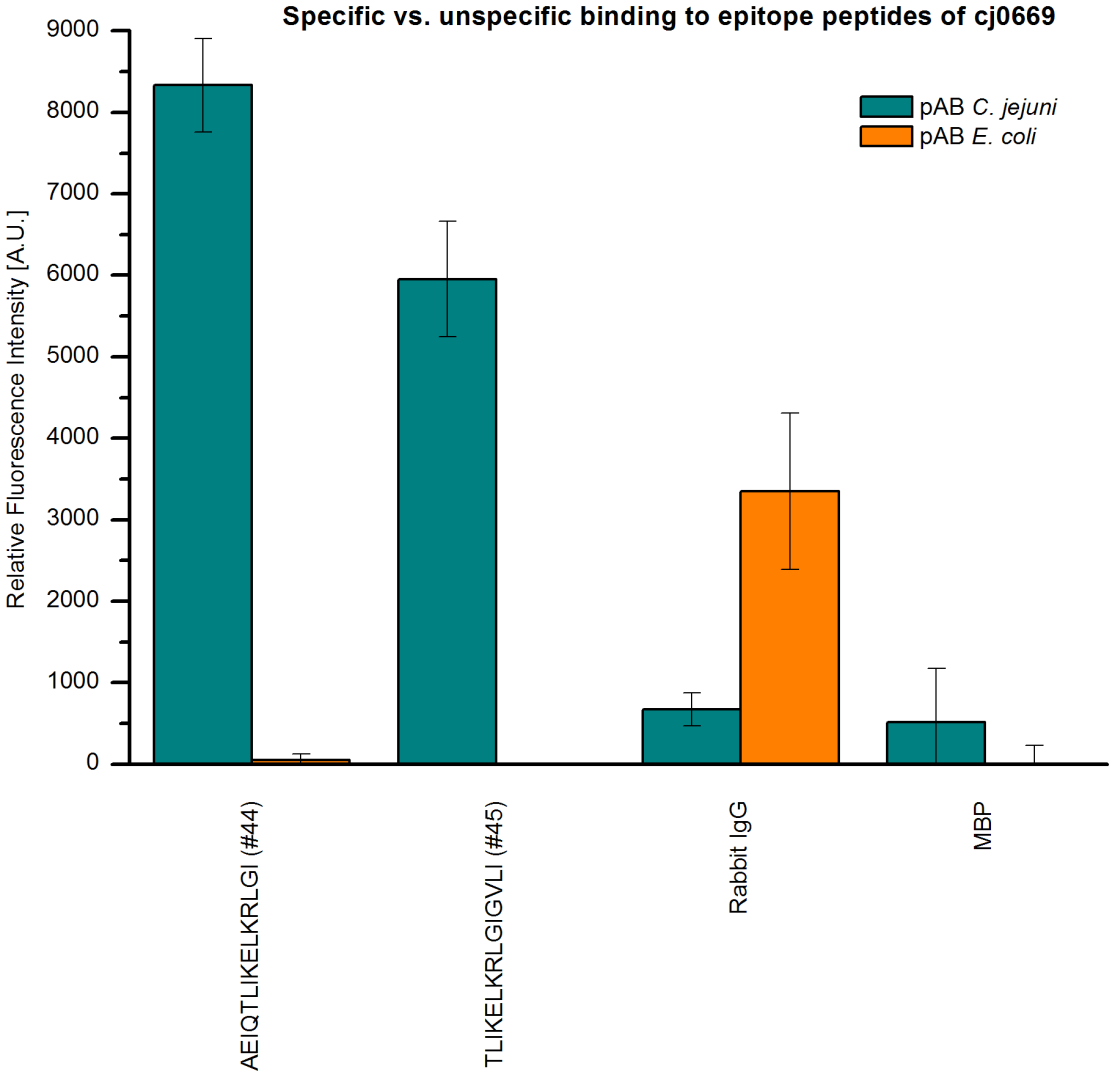
Specific vs. non-specific binding to epitope peptides of cj0669. The mean relative fluorescence intensities of peptides 44, 45, Rabbit IgG and MBP are compared after incubation with three different specific anti-*Campylobacter jejuni* antibodies to anti-*E.coli* antibodies. Error bars were calculated by propagation of uncertainty by Gauss. Peptides 44 and 45 of cj0669 show mean rfi values of approximately eight and six thousand A.U. respectively after incubation with polyclonal anti-*Campylobacter* antibodies. However, using *E.coli* antibodies the signals are almost gone amounting to less than 500 A.U. in rfi. This indicates a predominantly specific interaction of anti-*Campylobacter* antibody and peptides tested.

Cj0669, an ABC-transporter ATP-binding protein, showed significant intensities in two adjacent peptide spots as shown in Figure 2. The consensus sequence derived from these spots is TLIKELKRLGI, which is highly conserved in *C. jejuni* (see S2: TLIKELKRLGI is conserved in *C. jejuni)*. Next, we assessed specificity of these signals by applying antibodies reactive to other bacteria then *C. jejuni* in the epitope mapping to assess whether the binding occurs via the paratope of the antibody or in a non-specific manner. Figure 3 shows the results of these investigations. The mean rfis (n = 12) of peptide 44 and 45 are at 8000 and 6000 A.U. respectively after incubation with polyclonal antibody to *C. jejuni*, whereas these intensities drop below 50 A.U. if an *E.coli* antibody is used.

The remaining proteins painted a different picture. For cj1575 (S3:Epitope Mapping of cj1575) - a fragment with only 75 residues - no linear epitope was identified. The same was true for cj0623 (S4:Epitope Mapping of cj0623), cj0476 (S5:Epitope Mapping of cj0476) as well as cj1723 (S6:Epitope Mapping of cj1723) which harboured no linear epitopes. For the protein cj1380 (S7:Epitope Mapping of cj1380) only one peptide showed signal intensities above the positive control. Another ABC-transporter protein, cj0920c, possesses several regions, where signal intensities were above those of the positive controls, namely peptides 6 to 8 (aa 21 – 43), 17 to 19 (aa 65 – 87) as well as 56 and 57 (aa 221 – 239), see S8: Epitope Mapping of cj0920c.

The three potential antigenic regions were further assessed using transmembrane prediction tool TMHMM2.0 and the antigenic prediction tool by EMBOSS. Here, peptides 6 – 8 showed a consensus sequence, namely SPFAVWKFLDAL, which ought to be presented extracellular as well as being antigenic according to the prediction tools. For the other two sites, either no antigenicity was predicted or most of the amino acids lie within a transmembrane region. This is true for amino acids 47 – 69, 90 – 112 and 214 – 236. For a summary of the predicted characteristics, see S9: Transmembrane and antigenic potential of three potential epitope sites for cj0920c.Further, specificity control assays revealed that these signals do not drop significantly when using antibodies to *Salmonella enterica* indicative of non-specific binding to occur, see S10: Specific vs. non-specific binding to potential linear epitopes of cj0920c.

### Alanine Scan of consensus sequence TLIKELKRLGI of cj0669

**Figure 4:**
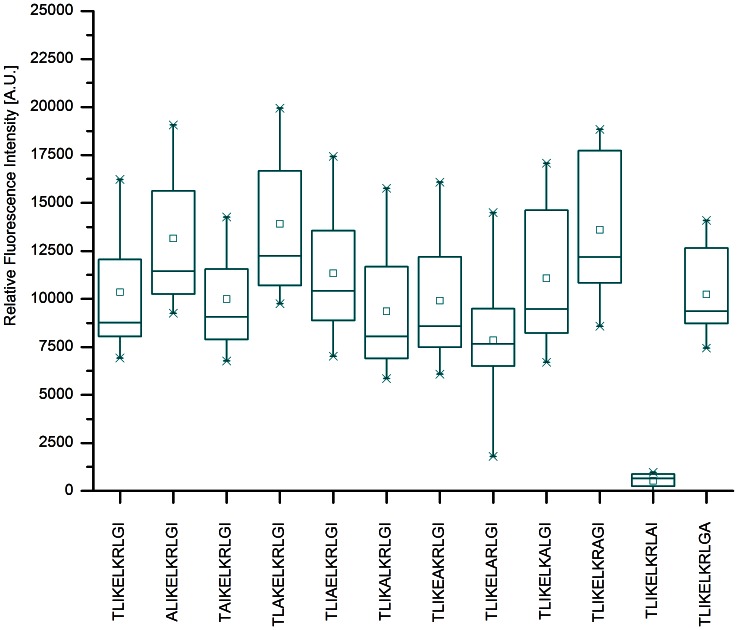
Alanine Scan of consensus sequence of cj0669 linear epitope. The Box-whisker-plot (n = 15) of the eleven residue long consensus peptide sequence of a cj0669 linear epitope after incubation with three different anti-*Campylobacter* antibodies. Each box represents 50% of the values, while 98% fall within the whiskers. The median is represented by a horizontal line within each box and the small rectangle corresponds to the mean of each sample. The original sequence TLIKELKRLGI shows a mean fluorescence intensity of about 10000 A.U. For most amino acids within this sequence, replacement by alanine has no to little effect. However, if glycine is replaced by alanine the peptide loses most of its binding activity, clearly seen by a drop of mean rfi to less than 1000 A.U. This equals a drop of more than 90% in intensity.

**Figure 5:**
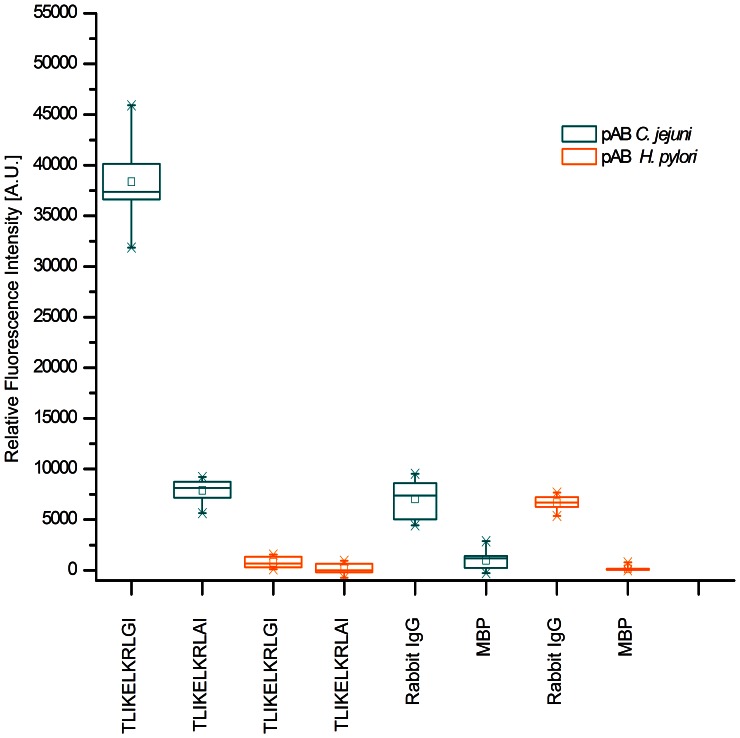
Binding specificity assay of epitope sequence in alanine scan. Box-whisker-plot (n = 12) of the eleven residue long consensus peptide sequence TLIKELKRLGI and its derivative TLIKELKRLAI of cj0669 after incubation with antibodies reactive to *C. jejuni* (green) and *H. pylori* (blue). Each box represents 50% of the values, while 98% fall within the whiskers. The median is represented by a horizontal line within each box and the small rectangle corresponds to the mean for each sample. For references the rfi values of the positive control, Rabbit IgG, and negative control, MBP, are indicated on the right for both antibody incubations. For the original sequence a mean value of 38000 A.U. can be observed after incubation with antibody to *C. jejuni*. This dropped to less than 8000 A.U. after alanine substitution. This represents a drop of roughly 80% in overall intensity. In contrast, neither original nor substituted peptide showed any significant mean values after incubation with the anti-*H. pylori* antibodies indicating specific binding to the peptide by the anti-*Campylobacter* antibody.

The influence of each amino acid within the consensus sequence TLIKELKRLGI was assessed by performing an alanine scan. As Figure 4 shows, the majority of amino acids in the sequence do not confer a change in binding intensity if replaced by alanine. However, in case of glycine a substantial drop in rfi values to less than 10% of the original value is observed. This indicates an important role of the glycine residue in the binding of the antibody. Further, these results were highly reproducible in a second set of experiments and the specificity of the binding was evaluated using antibodies reactive to *Helicobacter pylori* as summarized in Figure 5.

The green boxes on the left represent the values of original sequence TLIKELKRLGI and altered sequence TLIKELKRLAI after incubation with polyclonal antibodies to *C. jejuni*. As was observed in the previous figure, the drop in intensity is significant from a mean value of approximately 38000 A.U. to less than 7900 equal to roughly 80% decrease in the signal. In comparison, the adjacent boxes represent the data of the original sequence as well as TLIKELKRLAI after incubation with antibodies reactive to Helicobacter pylori (orange).Here, no significant difference between unaltered and altered sequence could be observed. Furthermore, the mean intensities were below 800 A.U. Thus, binding of an anti-*Helicobacter pylori* antibody to the sequence is approximately 2% as compared to the original binding of polyclonal antibodies to Campylobacter jejuni against this linear epitope. The same trend was observed when using antibodies reactive to *S. enterica* (S11: Binding specificity assay of TLIKELKRLGI with anti-*Salmonella* antibodies). This indicates low non-specific binding.

### Structural Modelling and Accessibility of the epitope

Modelling of cj0669’s structure was performed by SWISS-MODEL using automated mode. The template chosen during automatic identification was pdb1ji0A from the RCSB Protein Data Bank [35], which referred to the Crystal Structure Analysis of the ABC transporter from *Thermotoga maritim*a [36].

**Figure 6:**
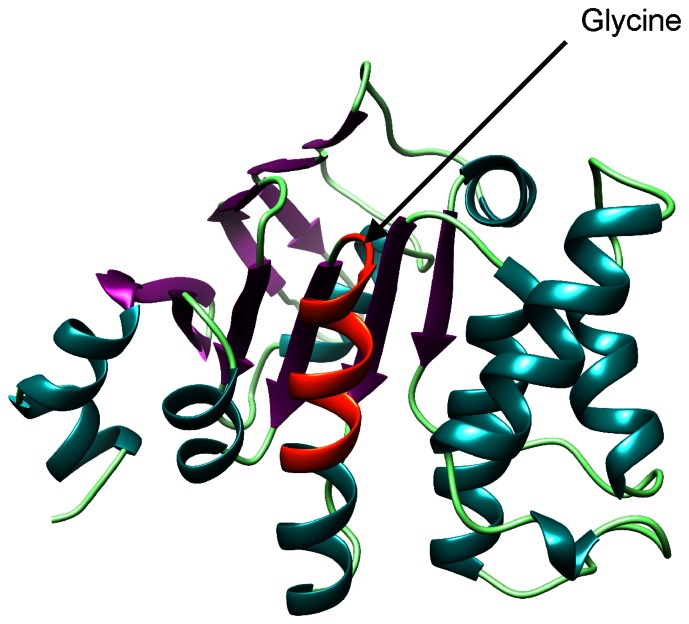
Structure of cj0669 based on SWISS-MODEL calculations. The structure of cj0669 was calculated using the SWISS-MODEL workspace automated method provided by Expasy (University Basel). The helices are shown in dark green, sheets in purple, coils in light green and the epitope site highlighted in orange. The sequence TLIKELKRLGI is part of an alpha-helix adjacent to a loop structure which connects the helix to a beta sheet. The glycine residue identified in alanine scanning to be of high relevance lies within the loop structure.

**Figure 7:**
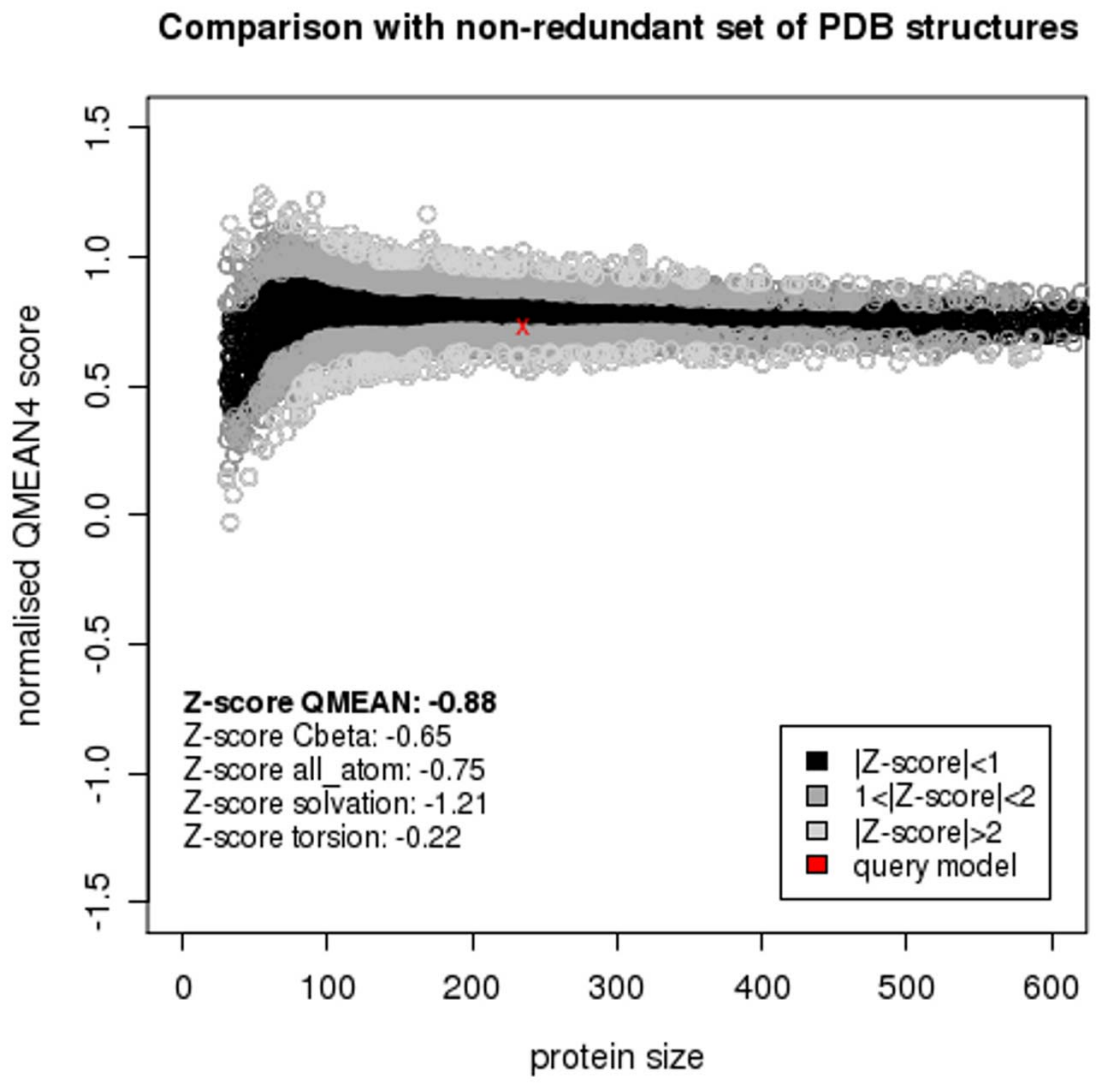
Query model z-value in comparison with set of PDB structures. The z-value is given as –0.88 and broken down in four different categories. The diagram shows the position of the query model (red cross) as compared to a non-redundant set of PDB structure dependent on the size of the protein. The current query model falls within the black area, indicating a model of high accuracy.

The model spans amino acids 3 to 236 of the molecule incorporating more than 90% of the residues. Figure 6 shows a graphical display of the structure with the distinct secondary structures highlighted. In addition, the epitope sequence TLIKELKRLGI is marked in orange. The first nine residues comprise parts of an alpha helix, whereas glycine and isoleucine form a loop to attach the alpha helix with a beta strand further down the primary sequence of the protein. The modelling was supported by statistical analyses including determination of a z-value [37] based on published data in the Protein Data Bank. Figure 7 shows the accuracy of the model based on the z-value calculations.

### Predicted characteristics of peptide TLIKELKRLGI

We analyzed the consensus peptide sequence TLIKELKRLGI by Geneious Pro 5.6.5. Notably, when predicting secondary structure and antigenic regions by EMBOSS garnier and antigenic tool respectively, the results matched the previous observations. An alpha helix precedes a loop that is connected to a beta-sheet. The only difference to the structural modelling by SWISS MODEL is the number of residues within the loop as EMBOSS only includes the glycine residue in the loop. For antigenic potential, EMBOSS antigenic predicts all amino acids within the consensus sequence to be part of antigenic sites except for lysine.

## Discussion

The development of a cDNA derived expression library and subsequent screening of the expressed fusion proteins has revealed several novel immunodominant proteins. Optimization during library construction by reducing the amount of rRNA molecules via DSN treatment has proven to greatly reduce the amount of rRNA clones as was shown for other applications elsewhere [38], [39]. This enhances the number of clones carrying mRNA-derived cDNA, thus increasing the number of clones potentially expressing proteins of interest.

For screening purposes antibodies were used, which were generated by immunizing rabbits with whole and partly lysed cells of *C. jejuni*. Therefore, both membrane-associated as well as soluble proteins were available during immune response. Thus, it was to be expected that the antibodies used in screening target both membrane and cytosolic proteins, which could be observed in the proteins identified. The screening not only detected the 22 proteins listed here but included other already known immunogenic proteins. These were excluded from further analyses as the main goal of this work was to identify novel proteins and their linear epitopes.

The screening and analysis of immunodominant behaviour by microarrays was highly reproducible. Some of the proteins showing the strongest signals during screening and microarray experiments were discarded, as they possess strong homologies within a wide spectrum of organisms. However, we focused on proteins specific for *Campylobacter* due to the potential application in a diagnostic tool. Thus, linear epitope mapping was performed on a subset of seven proteins. This revealed linear epitopes for two proteins, cj0669 and cj0920c, both ABC-type transporters, which were expected to be immunogenic [40]. The identified region TLIKELKRLGI of cj0669 proved to be specific showing little to none non-specific interactions with other antibodies. Most notably, *H. pylori* antibodies did not reveal any binding to the linear epitope. In the wake of the other antibodies tested, i.e. anti-*E.coli* and anti-*S. enterica*, this strongly suggests non-specific binding to be minute. Furthermore, the applicability of TLIKELKRLGI as a specific target for *Campylobacter* identification, antibody or vaccine production seems plausible.

Alanine scan has further identified the glycine residue to be of utmost importance for the antibody binding as replacing it by alanine reduces the intensity by at least 80%. This has been somewhat surprising as glycine is a non-charged, achiral residue. However, the presence of glycine might cause the protein to turn more easily provided by the glycine’s flexibility and consequently leading to a better accessibility. Thus, immunogenic ability might be rendered by the presence of glycine. In fact, epitopes containing glycine as an important residue have been reported before [41]–[43].

Next, we modelled the 3-dimensional structure of cj0669 in order to evaluate the accessibility of the potential epitope. This is an important feature when bearing a diagnostic application in mind. From the model, we could conclude that the glycine residue and the linear epitope are most likely presented as part of a loop structure adjacent to the end of an alpha helix. These are located on the outer rim of the protein, hence easily accessible for antibody binding to occur. Although, the structural information is merely based on modelling, the z-values of this model fall within close proximity to zero (–0.88) commonly associated with x-ray crystallographic results [37]. Consequently, the probability of this model to match the real structure is high. These findings are supported by further calculations performed using the EMBOSS garnier and EMBOSS antigenic algorithms. These results underline the antigenic potential of the consensus sequence TLIKELKRLGI as well as predicting almost identical secondary structures as the automated SWISS MODEL. Moreover, the secondary structure prediction changes when glycine is replaced by alanine, which further underlines the important role glycine most likely plays in antibody binding due to its ability to create a loop structure which would otherwise be absent with high probability.

In conclusion, TLIKELKRLGI seems a suitable candidate to be studied further and shows high potential for applications in a diagnostic tool or vaccination due to its good accessibility and antigenicity. This gains further momentum as TLIKELKRLGI is highly conserved in *C. jejuni,* whereas other species of *Campylobacter*, e.g. *C. coli*, *C. upsaliensi*s or *C. lari* possess other amino acid residues within this region. For *Helicobacter pylori,* this effect is even more pronounced. Specifically, the glycine residue at the tenth position that was revealed paramount for the binding is not present in *Helicobacter pylori,* see S2. Thus, TLIKELKRGLI seems a suitable candidate for specific diagnostic applications targeting *C. jejuni*.

The analysis of the remaining six proteins revealed linear epitopes only in the case of cj0920c, another ABC-type transporter. The region encompassing amino acids 27 – 38 SPFAVWKFLDAL seems to be the most promising as its rfi values are high in microarray experiments. Furthermore, the prediction tools determined this sequence to be antigenic and located extracellularly, thus easily accessible. This is not true for the other two regions detected from cj0920c, which are either lacking antigenic potential or are located within the cell or in transmembrane regions. Still, the results indicate non-specific binding to contribute mainly to the positive signal. This exempts SPFAVWKFLDAL from a suitable diagnostic application; however, further analysis might be helpful to investigate the full potential of this sequence as a specific epitope.

Although the analyses of full-length proteins on microarrays hint at the presence of antigenic sites within each protein, a lack of linear epitopes was observed. However, this was expected, as most naturally occurring epitopes are conformational and not linear [44]–[47]. The current method used for epitope mapping cannot detect conformational epitopes. Still, the presence of linear epitopes and their detection are important features in serological applications. This is particularly true for the high-throughput analysis of sera on microarrays as described elsewhere [48], [49].

The present work has accomplished two main goals. First, we have been able to identify a previously unknown antigenic site TLIKELKRLGI of cj0669 from *C. jejuni* and were able to determine the important residue involved in antibody binding as well as modelling the epitope’s accessibility within the full-length protein. For *C. jejuni* the generation of monoclonal antibodies against cj0669 is mandatory to further investigate the affinity and kinetics of the observed binding event by BIAcore. This might grant further insight whether or not this sequence is a suitable candidate for specific detection in a biosensor or if cj0669 is a suitable vaccine candidate. Mutagenesis of cj0669 might help to illuminate the function of this protein and its potential role if any in pathogenicity. Once this is determined, a monoclonal antibody might be used to cocrystallize the antigen for X-ray crystallography to assign a measured structure to the predicted model. On top of that, antibody validation by ELISA with a wide array of *C. jejuni* isolates is needed to evaluate the applicability of the derived antibody for future clinical point-of-care devices.

Additionally, next-generation sequencing of transcriptomes of a broad spectrum of *C. jejuni* strains ought to be beneficial in analyzing the presence and expression of the protein. This might provide further insight into the suitability of the protein for clinical applications and vaccine development. Consequently, it could help to identify potential sequence homologies or discrepancies, which as of now are limited to already published datasets. Thus, next-generation sequencing might be an attractive procedure to complement the approach presented herein.

**Figure 8:**
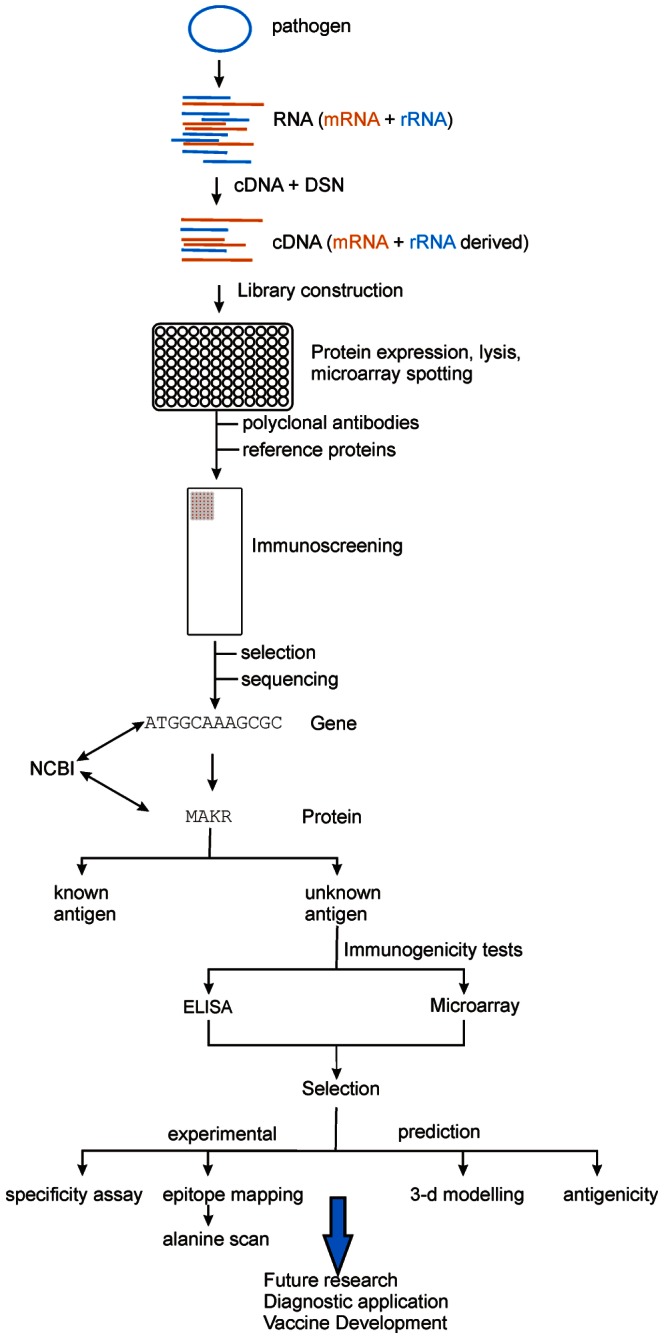
Schematic summary of the methods involved for library construction, screening and characterization. A bacterial pathogen is selected and its RNA isolated. After cDNA generation, normalization is performed to minimize the number of rRNA derived clones. Next, screening is performed using polyclonal antibodies and potentially immunogenic proteins are selected. The corresponding clones are sequenced and the genes and proteins identified, either by exact match (sequenced strain) or homology (clinical isolate). A set of candidate proteins is selected focusing only on previously unknown antigens, while known antigens are discarded. Immunodominant nature of each full-length protein is assessed by ELISA and microarray analyses to further narrow down the number of proteins to be tested via epitope mapping, if desired. Linear epitope mapping reveals potential antigenic sites, which are tested for specificity and in alanine scan. Finally, bioinformatic tools are used to model the 3-dimensional structure, accessibility and antigenicity of each protein. Afterwards, the most promising candidates can be used in future applications including but not limited to monoclonal antibody generation, kinetic measurements, structural and functional analyses and diagnostic applications.

Secondly, we have established and applied a quick and easy method for the screening of cDNA expression libraries in order to rapidly detect novel immunodominant proteins of bacteria, see Figure 8 for a summary.

This approach is easily transferable to other bacterial pathogens such as Methicillin-resistent *Staphylococcus aureus*, *Klebsiella pneumonia*, *Neisseria gonorrhoea*, *Pseudomonas aeruginosa*, *Salmonella enterica* and others. The information needed prior to analysis is minute, yet using fully sequenced strains is advantageous as it speeds up the identification of genes and proteins. Still, even unsequenced strains such as clinical isolates can easily be analyzed and should not pose any hindrance, as homologous proteins ought to be identified via BLAST. The more important prerequisite for the screening is the availability of polyclonal antibodies or patient sera. In the context of a diagnostic tool to be used in hospitals, patient sera are beneficial, as they resemble the desired immune response better, thus narrowing down the results to the clinically most relevant proteins. Finally, suitable references are useful, yet in most cases, some immunodominant antigens have already been described. Regardless, even without a suitable known antigen, this method could identify novel immunoreactive proteins and their linear epitopes with some minor adjustments to the overall calculations.

In conclusion, we have successfully shown the application of this method while gaining insight into some novel immunodominant proteins of an important gut pathogen, *C. jejuni.*

The current research emphasizes the need for future investigations in two distinct areas. First, more insight into the newly identified proteins of *C. jejuni* might foster the understanding of this enigmatic pathogen and help to illuminate its pathogenicity while providing suitable means for rapid detection and combating its spreading. Second, transferring this method to other bacterial pathogens will help to discover other immunodominant proteins, potentially leading to a broad spectrum of clinical applications and serological assays.

Additionally, it might be preferable to simplify the identification of conformational epitopes as this could greatly enhance the retrieval rate of suitable antigenic sites. The state-of-the-art technique involves the cocrystallization of an antigen with a monoclonal antibody [50], [51]. Besides, mutation analysis [52], mass-spectrometry [53] or CLIPS technology [54] are other methods to identify structural epitopes. However, the existing methods are extremely costly, time-consuming and demand high material inputs. Therefore, advances to simplify structural epitope detection are essential and would be ideally suited to be combined with our current approach.

Nevertheless, linear epitopes provide an important tool for clinical applications. While their low abundance poses a problem, their simplicity grants major benefits. They are rapidly synthesized and modified. This enables them to be used in a broad spectrum of assays. In a clinical context where antibody titer determination of patient sera is necessary, short peptides are extremely valuable. Production costs using chemical synthesis are relatively low and easier than recombinant expression of full-length proteins. Furthermore, specific peptides of pathogenic bacteria remove the need to use the entire pathogen, thus reducing the associated risk.

Although prediction-based strategies for antigens and linear epitopes have been published [55]-[58], their accuracy is often lacking [59]. In contrast, our approach offers an attractive dual procedure as it rests on experimental data that are complemented by a widespread support of bioanalytical tools. Thus, such a thorough approach facilitates to focus on relevant epitopes quickly, while rapidly evaluating their suitability for prospective diagnostic applications as compared to prediction-based or experimental methods alone.

Consequently, we believe our present findings to be of outstanding interest for diagnostic applications and to pave the way for future implementation. Moreover, the ability to quickly generate cDNA libraries and identify novel immunodominant proteins independent of the bacterium used should lay the foundations for future research with highly relevant pathogens. Creating a method to extend the detection to structural epitopes would add another tier to the current approach and greatly enhance the knowledge about antigenic sites. Further, we are expanding our focus to other pathogens to help elucidate novel antigens within a wide array of clinically relevant bacteria.

## Methods

### Bacterial strain

The strain *C. jejuni* NCTC 11168 was grown on solid Karmali media for 48 h at 37 °C under microaerophilic conditions (85% N_2_, 5% O_2_ and 10% CO_2_) within a hypoxic workstation (Coylabs). For RNA isolation, a liquid culture was prepared by inoculating 10 ml of brain-heart-infusion broth (BHI) with a single colony and incubated overnight at 37°C, 140 rpm under microaerophilic conditions. This overnight culture was used to inoculate a flask containing 100 ml fresh BHI medium and incubated for 16 h prior to harvesting.

### Antibodies

For initial screening, a Rabbit polyclonal IgG antibody to *C. jejuni* (Acris AP24002PU-N) was used. For further microarray analyses of a subset of candidate proteins, ELISA, epitope mapping and alanine scanning this antibody was used as well as two other Rabbit polyclonal IgG antibody to *C. jejuni* (Abcam ab22542 and Abd Serotec 1744-9035). The immunogen used for generation of antibodies to *C. jejuni* was *C. jejuni* ATCC 29428. For specificity assays Rabbit polyclonal IgG antibody to *E.coli* BL21 (MicroMol #322), *S.enterica* (Abcam ab35156), *S. aureus* (Fitzgerald 20C-CR1274RP) and *H. pylori* (Abcam ab20459) were used respectively. Detection was achieved by usage of secondary antibodies. A Goat polyclonal to Rabbit IgG conjugated with Chromeo™-546 (Abcam ab60317) for fluorescent and conjugated with Horseradish peroxidase (Abcam ab6721) for a colorimetric readout was applied.

### Harvesting of cells, lysis and total RNA extraction

The cells were harvested by centrifugation (2000 x g, 10 min). The supernatant was discarded and the pellets resuspended in fresh BHI medium. Subsequently, 0.5 ml of the bacterial suspension were added to 1 ml of RNAprotect Bacteria Reagent (Qiagen) in a 2 ml tube, vigorously vortexed for 5 s and incubated for 5 min at room temperature. After centrifugation (5000 x g, 10 min) the pellets were resuspended in 200 µl of lysis buffer (30 mM Tris-Cl, 1 mM EDTA, 15 mg/ml Lysozyme, >12 mAU Proteinase K) by pipetting and vortexing for 10 s. The solution was incubated for 10 min using a Thriller Thermomixer (Peqlab) at room temperature and 1000 rpm. After addition of 700 µl buffer RLT and 500 µl 96% Ethanol, the lysate was applied to RNeasy Bacteria Mini Kit spin columns (Qiagen) for RNA isolation following the manufacturer’s instructions. After loading of the lysate, an on-column DNase digest was performed using RNase-free DNase I solution (Qiagen) according to manufacturer’s instructions. The isolated total RNA was eluted in 50 µl of RNase-free water and its concentration and purity analyzed by Nanodrop (Peqlab) measurements.

### Analysis of RNA integrity

The quality of isolated RNA was assessed using the RNA 6000 Pico kit and Bioanalyzer 2100 (Agilent). The total RNA was diluted to a working concentration of 200 – 500 pg/µl. The analysis was performed following manufacturer’s instructions and the RNA integrity number (RIN) calculated by the 2100 Expert software (Agilent). The RIN is defined to fall into a range of 0 to 10, with a higher score indicating an intact RNA, whereas lower numbers are associated with degraded RNA molecules [60].

### Polyadenylation of total RNA

In order to use bacterial mRNA as a substrate in cDNA synthesis, polyadenylation was mandatory. The tailing was achieved using the Poly(A) Polymerase tailing kit (Epicentre) following the alternate protocol offered by the manufacturer. Briefly, up to 10 µg of total RNA were combined with 2 µl Poly(A) Polymerase reaction buffer, 2 µl 10 mM ATP, 0.5 µl Riboguard RNase Inhibitor and 1 µl Poly(A) Polymerase (4 U) in a total reaction volume of 20 µl. The reaction was incubated for 20 min at 37°C, terminated by the addition of 1 µl 0.5 M EDTA and purified by RNeasy Mini Kit (Qiagen) following manufacturer’s instructions. Yield and purity were determined by nanodrop measurements.

### cDNA synthesis

For cDNA synthesis, the In-fusion® SMARTer™ Directional cDNA Library Construction Kit (Clontech) was used according to manufacturer’s instructions with slight modifications. 3.5 µl total, polyadenylated RNA were mixed with 1 µl of 3′ In-Fusion SMARTer CDS Primer, heated first for 3 min at 72°C and then incubated for additional 2 min at 42°C. After addition of 5.5 µl Mastermix (2 µl 5x First Strand Buffer, 0.25 µl 100 mM DTT, 1 µl 10 mM dNTPs, 1 µl 12 µM SMARTer V Oligonucleotide, 0.25 µl RNase Inhibitor and 1 µl SMARTscribe™ Reverse Transcriptase) the tubes were incubated for 90 min at 42°C. The reaction was terminated at 68°C for 10 min.

For second strand cDNA synthesis two 2 µl aliquots of first strand reaction were used in long distance PCR using Phusion Polymerase (Finnzymes). Each PCR reaction was comprised as follows: 2 µl First-strand reaction, 70 µl RNase-free water, 20 µl 5x Phusion HF buffer and 2 µl each of dNTP mix (10 mM), 5′ PCR Primer II A (12 µM), 3′ In-Fusion SMARTer PCR Primer (12 µM) and Phusion Polymerase with a total reaction volume of 100 µl. The PCR reactions were subjected to the cycling program with 98°C for 1 min as initial denaturation followed by 15 cycles of 10 s denaturation at 98°C, 30 s of primer annealing at 65°C and 6 min extension at 72°C. For improved PCR results optimization was performed as follows; 30 µl of the 15 cycle experimental tube were transferred to a separate PCR tube, cycling commenced and aliquots of 5 µl each were collected after 15, 18, 21, 24 and 27 cycles total. The different cycles were compared by gel electrophoresis and the experimental tubes subjected to additional cycles if necessary. Finally, PCR reactions were purified using the QIAquick PCR Purification Kit (Qiagen). The purity and yield of each reaction were analyzed by nanodrop measurements.

### Normalization of cDNA

Normalization of double-stranded cDNA was achieved with the Trimmer-2 cDNA Normalization Kit (Evrogen) to reduce the number of cDNA molecules derived from rRNAs. Briefly, 12 µl of cDNA (approx. 100 ng/µl) were mixed with 4 µl of 4x Hybridization buffer. For the trimming reaction 4 µl of this mixture were distributed to four different PCR tubes and overlaid with a drop of PCR-grade mineral oil. After centrifugation (13000 x g, 2 min), the tubes were incubated for 2 min at 98°C followed by 5 h at 68°C. Next, pre-heated (68°C) Duplex-specific nuclease (DSN) master buffer was added to each tube and incubation prolonged for 10 min. DSN was added to the first three tubes in decreasing concentrations – 1 U/µl, 0.5 U/µl and 0.25 U/µl – with the fourth tube receiving DSN storage buffer and no enzyme as a control reaction. The incubation was continued for 25 min at 68°C. After addition of 5 µl DSN stop solution and subsequent incubation for 5 min at 68°C, the tubes were placed on ice. The chilled reaction was diluted by addition of 25 µl sterile, RNase-free water. For amplification of normalized cDNA, 1 µl of each reaction was used as template in PCR. Each PCR reaction contained 1 µl of template from the normalization reaction, 33 µl nuclease-free water, 10 µl 5x Phusion HF buffer, 1 µl 10 mM dNTP mix (NEB), 2 µl of each primer 5′ PCR Primer II A (12 µM), 3′ In-Fusion SMARTer PCR Primer (12 µM) and 1 µl Phusion Polymerase. The PCR was performed with initial denaturation at 98°C for 1 min and seven cycles of denaturation at 98°C for 10 s, primer annealing at 65°C for 30 s and extension at 72°C for 3 min, respectively. For optimization, the control tube was subjected to 7, 9, 11, and 13 cycles with 12 µl aliquots taken every two cycles. The optimization samples were analyzed by gel electrophoresis (1% agarose, TAE, 100 V) and the optimal cycle number determined. The remaining three tubes were subjected to 9 + X cycles with X being the differential of the optimized cycles to the originally performed seven cycles. After the second PCR, the experimental reactions were compared to the optimal control reaction using gel electrophoresis as above. Reactions showing a successful normalization were combined and used in a third PCR reaction. After the final PCR, the reactions were purified by QIAquick PCR Purification Kit.

### In-fusion cloning and cloning vector

For cloning pFN18A (Promega) was used as a vector, as it features a N-terminal encoded HaloTag® fusion protein, which allows for specific and covalent binding to a unique ligand, thus reducing background and minimizing cross-reactivity in immunoassays with HaloLink™ microarrays harbouring the ligand on its surface. First, the vector needed to be linearized to be used with the In-Fusion cloning technology. This was achieved by reverse PCR using IFS 18A for (5′ TTGATACCACTGCTTTTCCATGGCGATCGCGTTATC 3′) and IFS 18A rev (5′ TCTCATCGTACCCCGTGTTTAAACGAATTCGGGCTCG 3′). Each reaction contained 2 µl each of 1∶10 diluted pFN18A (10 ng/µl) and the two primers, 10 µl 5x Phusion HF buffer, 1 µl 10 mM dNTPs, 0.5 µl Phusion Polymerase and 32.5 µl nuclease-free water to reach a total reaction volume of 50 µl. The PCR was run using a 25 cycle two-step program with 98°C denaturation for 10 s and 4 min extension at 72°C. After completion, 2 µl of *Dpn*I (20 U/µl) were added to the reaction and incubated at 37°C for 1 h. The presence of a single band was checked by gel electrophoresis and the remaining reaction purified by QIAquick PCR Purification Kit. Cloning of normalized cDNA and linearized pFN18A vector was performed following the manufacturer’s instructions within the In-Fusion SMARTer Directional cDNA library construction kit (Clontech).

### Electroporation

2 µl of the cloning reaction were mixed with 25 µl of electrocompetent Acella™ *E.coli* cells (Mobitec), a BL21 derivative, and electroporated in 1 mm cuvettes using the EasyjecT Plus electroporator (Peqlab). Conditions for electroporation were as follows: voltage  = 1400 V, capacity  = 25 µF, resistance  = 200 Ω and pulse duration of 5 ms.

The electroporated cells were added to 970 ml of Super Optimal Broth with Catabolite Expression (SOC) and incubated at 37°C for 1 h with shaking at 250 rpm. Afterwards, 150 µl of the transformation reaction were plated on Lysogeny Broth (LB) Agar containing ampicillin. For each reaction, at least two plates were prepared and incubated at 37°C for 16 h.

### Selection of clones for screening, protein expression, lysis

A total number of 1536 clones were selected and transferred to 1.3 ml U96 DeepWell™ Plates (Nunc) containing 0.8 ml LB-amp. The plates were incubated overnight at 37°C, 130 rpm. On the next day, the DeepWell™ Plates were centrifuged, the supernatant discarded and the pellets resuspended in 370 µl of LB-amp. A new set of U96 DeepWell™ Plates was prepared with 850 µl of fresh LB-amp and inoculated with 100 µl each from the resuspended overnight cultures. The remaining 270 µl of resuspended overnight culture were mixed with 30 µl of sterile-filtered DMSO and stored at –80°C. The newly inoculated plates were incubated for 6 h at 37°C, 130 rpm. Afterwards, the temperature was reduced to 20°C, incubation continued for 1 h and protein expression induced by addition of 2 µl of 0.5 M β-D-1-thiogalactopyranoside (IPTG). Incubation persisted overnight at 20°C, 130 rpm. The cells were harvested by centrifugation (2500 x g, 10 min), the supernatant discarded and the pellets frozen at –20°C. After 15 min the pellets were resuspended in 180 µl of EasyLyse™ Bacterial Protein Extraction Solution (Epicentre) and incubated for 5 min at room temperature. DNase I was mixed with DNase reaction buffer (10 mM Tris-HCl, 2.5 mM MgCl_2_, 10 mM CaCl_2_), added to the reaction and incubation was carried on for 10 min at 37°C. The plates were centrifuged to collect cell debris for 3 min at 2500 x g. For each sample 10 µl of lysate were transferred to 384 microtiter plates (Genetixx), which were used as reservoirs for the spotting procedure.

### Immunoscreening using Halolink Microarrays

The samples were spotted onto HaloLink™ slides (Promega) using the QArray2 microarray spotter (Molecular Devices). 384 different samples were spotted per slide with three replicate slides per screening. In total 1536 samples were screened on 12 slides (n = 3). Each sample was spotted as quadruplicates with controls in two identical sets of eighteen 10×10 subarrays each (total number of spots per slide 3600). The controls used included HT-HisJ, HT-CjaA and HT-Peb1a as positive reference proteins as these have been described as immunodominant before. As specificity controls HT-ArgC and HT-PyrC were used, representing proteins without known immunodominant behaviour, thus binding of the polyclonal antibodies is not expected. In addition, two different *E.coli* strains – Acella™ electrocompetent cells and KRX single-step competent cells (Promega) – were spotted as further controls. As those two lack proteins expressed with a HaloTag®, they are used as negative controls.

After spotting of the samples, the slides were incubated for 1 h at room temperature in a humidity chamber. Next, slides were washed with PBS + 0.05% IGEPAL® CA-630 (PBSI, Sigma Aldrich) and dried by nitrogen flow. The 2 Well ProPlate™ module (Grace Biolabs) was attached to each slide. The top chamber was filled with 1.5 ml of rabbit-polyclonal antibody to *C. jejuni* (Acris, 2 µg/ml) in PBS. The bottom chamber was incubated with PBS only. After 2 h of incubation at room temperature with gentle rocking, both chambers of each slide were washed three times with 2 ml of PBSI. Secondary antibody (Goat-polyclonal to Rabbit IgG conjugated with Chromeo™-546, Abcam, 5 µg/ml) was subjected to each chamber in PBS and the slides were incubated at room temperature for 2 h in the dark under gentle rocking. Finally, slides were washed three times with PBSI, the ProPlate™ modules removed and the slides dried by nitrogen flow. The slides were scanned on an Axon Genepix 4200A laser scanner (Molecular Devices) with the following settings: 532 nm laser, PMT gain 400, 40% laser power, lines to average 1, 10 µm resolution and standard green emission filter at 575 nm.

### Microarray Analysis

The raw data sets of all the microarray analyses in this publication have been deposited in NCBI’s Gene Expression Omnibus [61] and are accessible through GEO Series accession number GSE44717. The median fluorescence intensity of each spot corrected by the local background (median F532 – B532) was used. Further, relative fluorescence intensity (rfi) was calculated by subtracting the signals of the bottom chamber from the raw data signals of the top chamber to account for non-specific binding of secondary antibodies. For screening of expression libraries we used the contrast method with either ArgC or PyrC as specificity control to determine the contrast via the formula:
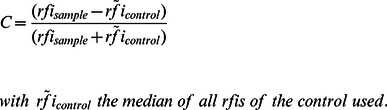



### Sequencing

Clones harbouring strong signals in microarray screening were selected to be sequenced. Sequencing was performed externally by LGC Genomics using HT7F (5′ ACATCGGCCCGGGTCTGAATC 3′) and FLXR (5′ CTTCCTTTCGGGCTTTGTTAG 3′) primers.

### Subcloning of positive candidates

After sequencing and identification of potentially immunodominant proteins, primers were designed to generate full-length clones for each identified gene, see S12 for a list of the primers used. Cloning was performed as mentioned above with slight modifications. The pFN18A vector was linearized using the following primer set; 18A IF linear for (5′ GTTTAAACGAATTCGGGCTC 3′) and 18A IF linear rev (5′ GGCGATCGCGTTATCGCTCTG 3′) with PCR conditions as mentioned before. Protein expression, lysis and spotting of full-length proteins were performed as described above. The slides were incubated for 1 h at room temperature in a humidity chamber. For incubation with antibodies 3 Well or 16 Well ProPlate™ modules (Grace Biolabs) were attached to the HaloLink™slides. Processing of the slides was done similar to the original screening, however several different antibodies were used, see section antibodies.

### ELISA

For testing of immunodominant characteristics with ELISA, the crude lysate was first purified using HaloLink™ magnetic beads (Promega) following the manufacturer’s instructions. The proteins of interest were subsequently cleaved off by digestion with ProTEV protease (Promega) and concentration was determined by nanodrop measurements. The samples were diluted to a total protein content of 20 µg/ml in PBS and 50 µl of each sample was added to MaxiSorb Plates (Nunc). Each sample was analyzed at least in triplicate. The ELISA plate was covered with a lid and incubated overnight at 4°C in a humidity chamber. After five washing steps each with PBS + 0.05% Tween-20 (PBST), the plates were blocked using 200 µl 5% non fat dried milk in PBS per well for 2 h. Afterwards, plates were washed three times with PBST. 100 µl of primary antibody solution (c = 4 µg/ml) in PBS containing 1% non fat dried milk were applied to each well using the respective desired antibody or PBS for controls. The plates were incubated for 2 h at room temperature and washed four times with PBST. Next, 100 µl of conjugated secondary antibody (Goat polyclonal to Rabbit IgG conjugated with Horseradish peroxidase, Abcam ab6721, c = 20 ng/ml) were added to each well and incubation carried on for 1 h. Finally, plates were washed once again four times with PBST and 100 µl 3,3′,5,5′-Tetramethylbenzidine (TMB, Sigma-Aldrich) was added to each well for detection. After 30 min of incubation at room temperature in the dark, the reaction was stopped by applying 100 µl of 2 M H_2_SO_4_ to each well. The optical density of each well was measured using the OMEGA Fluostar (BMG Labtech) at a wavelength of 450 nm.

### Bioinformatics

Primers were designed using Primer3 [62] within Geneious Pro 5.6.5 [63]. The sequenced inserts were identified by BLAST [64].Peptide sequence secondary structures were predicted using the EMBOSS garnier [65] algorithm and the transmembrane regions predicted by TMHMM2.0 [66], [67]. Antigenic sites were predicted by EMBOSS antigenic [68], [69]. Data evaluation was performed by OriginPro 8 G (Originlab) and Microsoft Excel. 3-dimensional structure predictions were performed using the SWISS MODEL automated mode [70]–[74] and pdb files were visualized and analyzed by the UCSF Chimera package [75]. Chimera is developed by the Resource for Biocomputing, Visualization, and Informatics at the University of California, San Francisco (supported by NIGMS P41-GM103311).

### Comparative data analysis

Analysis of full-length proteins was achieved by combining the results from ELISA and microarray data. Hence, the rfi of each sample was calculated. Next, a normalized rfi was generated by dividing the rfi of each sample by the median rfi of all the samples within an area of interest, i.e. incubation compartment. From these normalized rfis a median and standard deviation was calculated. If the median normalized rfi of the positive control was below the median normalized rfi of any of the negative references whilst considering the standard deviations, the test was rendered invalid. If the test passed the above criterion, the Q values were calculated as follows: 
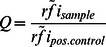



with 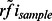 the median of normalized rfis of the sample and 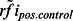 the median of the normalized rfis of the positive control HisJ respectively CjaA. The resulting error was calculated by error propagation according to Gauss. Finally, incorporating all valid tests, the mean Q value was determined along with its resulting error following error propagation by Gauss, see Table 1.

### Epitope Mapping

Several proteins were chosen for epitope mapping. These were the proteins encoded by cj0467, cj1723, cj0669, cj1380, cj0920, cj1575 and cj0623. The proteins were divided into 15-mer oligopeptides with an overlap of 11 amino acids *in silico*. The synthesis and coupling to microarray slides was performed externally by JPT Peptide Technologies GmbH. Each peptide sequence was applied 9 times to one slide. The slides were used with Proplate 3-Well Chamber system (Grace) allowing for incubation with different antibodies. First, the slides were blocked with SuperBlock blocking buffer (Thermo Fischer) for 2 h, washed five times with PBS + 0.05% Tween-20, primary antibodies applied, incubated overnight at 4°C with mild rocking, washed again, secondary antibodies applied for 2 h in the dark and after a final washing procedure, dried and scanned as above. Three different primary antibodies to *C.jejuni* were tested. The bottom chamber was always used as a control chamber, incubated only with secondary antibody.

### Alanine Scan

The peptide TLIKELKRLGI and 11 derivatives thereof substituting one amino acid for alanine were synthesized and coupled to microarray slides by JPT Peptide Technologies GmbH. Each peptide was applied 9 times to one slide. Incubation procedure was performed as described above for epitope mapping.

### SDS-PAGE

The expression of the desired HaloTag® fusion proteins was checked by SDS-PAGE. After lysis of cells, 2 µl of each protein extract was mixed with 1 µl of 10 µM HaloTag® Alexa 488 ligand. After addition of 7 µL 1x TBS (100 mM Tris, 150 mM NaCl, pH 7.6) the reaction was incubated at room temperature for 30 minutes. 2 µl of each reaction were removed, mixed with 8 µl of 5x loading buffer (Fermentas) and 1 µl DTT and heated for 5 min at 70°C. The separation was performed on a Mini-Protean TGX Gel (Biorad, any kD, 15 wells) in a Protean II xi Cell chamber (Biorad) for 30 min at 200 V. As a size reference Benchmark Fluorescent Protein standard (Life Technologies) was used. Fluorescence was measured in a FLA-5100 (Fujifilm) with excitation at 473 nm.

## Supporting Information

Figure S1 **RIN.** Electropherogramm and RNA Integrity number (RIN) for sample 5, a total RNA isolated from *C. jejuni* NCTC 11168, after analysis using the RNA 6000 Pico kit and the Agilent Bioanalyzer 2100. The RIN equals 9 and the ratio of 23S to 16S rRNA is 1.8. On the right hand, a virtual gel picture is presented as calculated by the Agilent Expert 2100 software.
(TIF)

Figure S2 **LIKELKRLGI is conserved in** ***C. jejuni*** **.** The protein sequence of Cj0669 was BLASTed and subsequently aligned according to a BLOSUM62 matrix. As a reference sequence TLIKELKRLGI of Q0PAK5, the protein encoded by cj0669 from *C. jejuni* NCTC 11168 was used. The dots indicate an agreement to the reference, while differences are given by the one letter amino acid code. The first 13 sequences including the references are 100% identical and are all derived from *C. jejuni* proteins. For other species of Campylobacter such as *C. coli* or *C. upsaliensis* several of the residues are replaced. For other organisms, especially *Helicobacter pylori* the degree of replaced residues increases. The positions showing the most conservation throughout are residues 3, 6, 9 and 11. However, residue 10, the glycine which was revealed to be paramount for the binding of the antibody is not found in *Helicobacter pylori* proteins.
(PDF)

Figure S3 **Epitope Mapping of cj1575.** Box-whisker-plot (n = 15) showing the relative fluorescence intensities of the different overlapping peptides including Rabbit IgG (red) and MBP (blue) as positive and negative controls. Each Box represents 50% of the values, while the whiskers enclose 98% of the data. The median is indicated by a horizontal line and the mean represented by a small rectangle.
(TIF)

Figure S4 **Epitope Mapping of cj0623.** Box-whisker-plot (n = 15) showing the relative fluorescence intensities of the different overlapping peptides including Rabbit IgG (red) and MBP (blue) as positive and negative controls. Each Box represents 50% of the values, while the whiskers enclose 98% of the data. The median is indicated by a horizontal line and the mean represented by a small rectangle.
(TIF)

Figure S5 **Epitope Mapping of cj0476.** Box-whisker-plot (n = 12) showing the relative fluorescence intensities of the different overlapping peptides including Rabbit IgG (red) and MBP (blue) as positive and negative controls. Each Box represents 50% of the values, while the whiskers enclose 98% of the data. The median is indicated by a horizontal line and the mean represented by a small rectangle.
(TIF)

Figure S6 **Epitope Mapping of cj1723.** Box-whisker-plot (n = 12) showing the relative fluorescence intensities of the different overlapping peptides including Rabbit IgG (red) and MBP (blue) as positive and negative controls. Each Box represents 50% of the values, while the whiskers enclose 98% of the data. The median is indicated by a horizontal line and the mean represented by a small rectangle.
(TIF)

Figure S7 **Epitope Mapping of cj1380.** Box-whisker-plot (n = 15) showing the relative fluorescence intensities of the different overlapping peptides including Rabbit IgG (red) and MBP (blue) as positive and negative controls. Each Box represents 50% of the values, while the whiskers enclose 98% of the data. The median is indicated by a horizontal line and the mean represented by a small rectangle.
(TIF)

Figure S8 **Epitope Mapping of cj0920c.** Box-whisker-plot (n = 15) showing the relative fluorescence intensities of the different overlapping peptides including Rabbit IgG (red) and MBP (blue) as positive and negative controls. Each Box represents 50% of the values, while the whiskers enclose 98% of the data. The median is indicated by a horizontal line and the mean represented by a small rectangle. Several parts of the protein show intensities above the positive control, namely peptides 17 to 19, 56 and 57 as well as peptides 6 to 8. This indicates potential antigenic sites within the above peptides.
(TIF)

Figure S9 **Transmembrane and antigenic potential of three potential epitope sites for cj0920c.** Shown are the three regions represented by peptides 6–8 (aa 21–43), 17–19 (aa 65–87) and 56–57 (aa 221–239). Antigenic regions were predicted by EMBOSS antigenic with a minimum size of 5 residues. Transmembrane regions were predicted using TMHMM2.0. For the sequence SPFAVWKFLDAL both antigenic site and extracellular position are predicted, while the other amino acids are either not antigenic or located within the cytoplasm or transmembrane regions.
(TIF)

Figure S10 **Specific vs. non-specific binding to potential linear epitopes of cj0920c**. The bars represent the mean values (n = 15) of rfi values after incubation with polyclonal antibody to *C. jejuni* (green) and *Salmonella enterica* (orange). The mean values for each peptide fall within the same range or possess overlapping standard deviations. Thus, no specific interaction of the antibody to the epitope is present; rather a non-specific binding seems likely.
(TIF)

Figure S11 **Binding specificity assay of TLIKELKRLGI with anti-** ***Salmonella*** **antibodies.** The different sequences tested in alanine scanning are shown in the box-whisker-plot (n = 15) with each box representing 50% of the values. The whiskers encompass 98% of the values, the median is indicated by a horizontal line and the mean represented by a small rectangle. The 12 boxes in green on the left represent the results after incubation with polyclonal antibody to *S. enterica.* For comparison, the two red boxes show the original signals from Fig. 4 for the sequence TLIKELKRLGI as well as TLIKELKRLAI, after incubation with polyclonal antibodies to *C. jejuni*. All the green boxes fall into the same range as the altered sequence TLIKELKRLAI, where alanine replaced the glycine residue, which possessed only 10% intensity of the original sequence. Thus, no specific interaction of the antibody to the epitope is present; rather a non-specific binding seems likely.
(TIF)

Figure S12 **Primers used in this study.** The name of each primer, the corresponding target gene or vector and its sequence in 5′ to 3′ direction is given. For each gene, F represents the forward primer and R the reverse. The primers were used for cloning in the In-Fusion SMARTer™ directional cDNA library construction kit.
(XLS)
