# Correction: Rapid Identification of Novel Immunodominant Proteins and Characterization of a Specific Linear Epitope of *Campylobacter jejuni*

**DOI:** 10.1371/annotation/94f7cbba-f1b3-4a65-b1d5-6c35c04fa201

**Published:** 2013-07-01

**Authors:** Sebastian Hoppe, Frank F. Bier, Markus v. Nickisch-Rosenegk

The name of the third author was given incorrectly. The correct name is: Markus von Nickisch-Rosenegk. 

The correct citation is: Hoppe S, Bier FF, von Nickisch-Rosenegk M (2013) Rapid Identification of Novel Immunodominant Proteins and Characterization of a Specific Linear Epitope of Campylobacter jejuni. PLoS ONE 8(5): e65837. doi:10.1371/journal.pone.0065837. 

The abbreviation in Contributions correctly reflects the name as it is. 

