# Supplementary figures and images for: Rapid Identification of Novel Immunodominant Proteins and Characterization of a Specific Linear Epitope of Campylobacter jejuni
Source: PLoS One. 2013 May 29;8(5):e65837. doi: 10.1371/journal.pone.0065837 (PMC3667084; doi:10.1371/journal.pone.0065837)

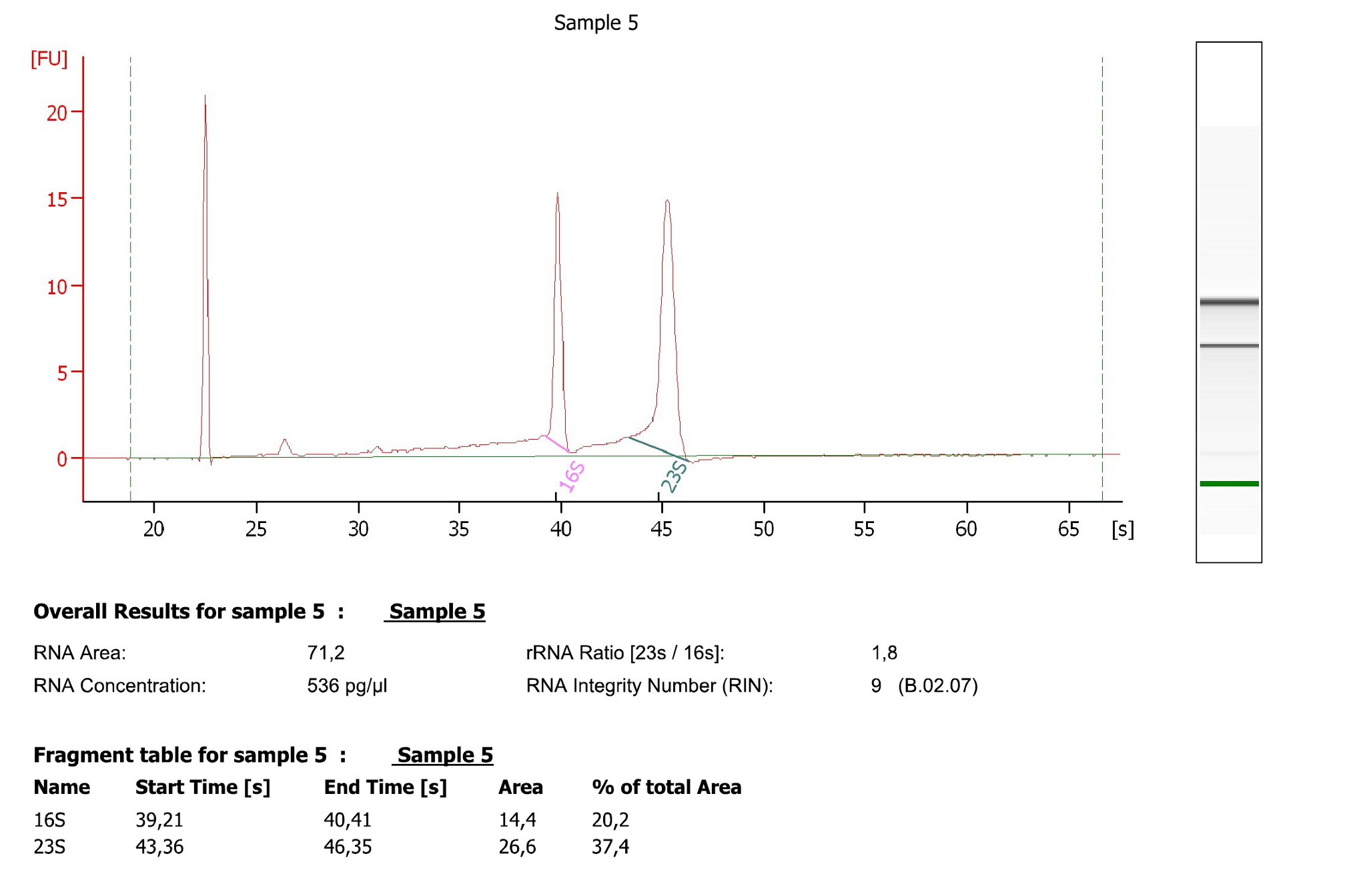

Supplement: Figure S1 — RIN. Electropherogramm and RNA Integrity number (RIN) for sample 5, a total RNA isolated from C. jejuni NCTC 11168, after analysis using the RNA 6000 Pico kit and the Agilent Bioanalyzer 2100. The RIN equals 9 and the ratio of 23S to 16S rRNA is 1.8. On the right hand, a virtual gel picture is presented as calculated by the Agilent Expert 2100 software. (TIF) [file pone.0065837.s001.tif]

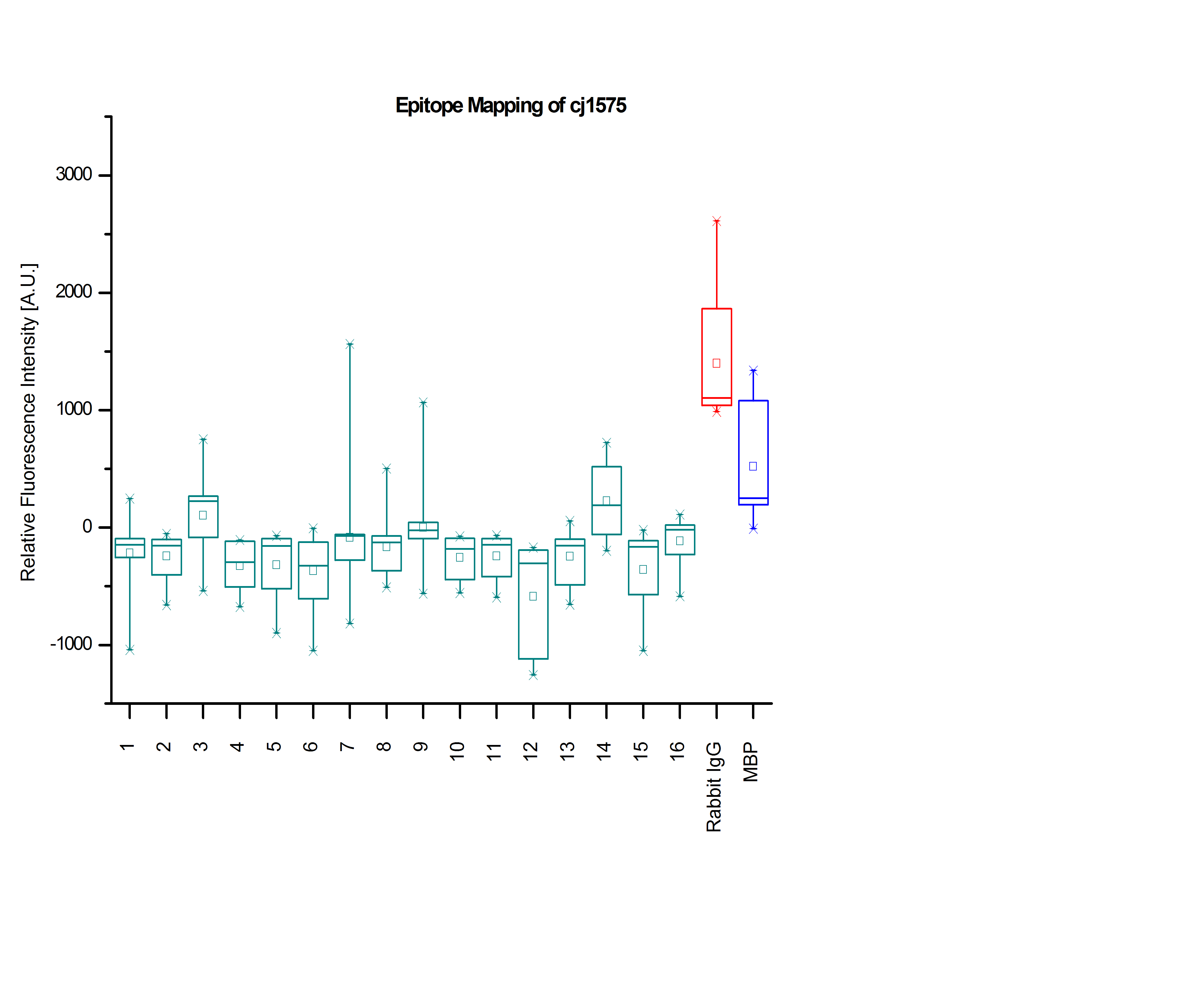

Supplement: Figure S3 — Epitope Mapping of cj1575. Box-whisker-plot (n = 15) showing the relative fluorescence intensities of the different overlapping peptides including Rabbit IgG (red) and MBP (blue) as positive and negative controls. Each Box represents 50% of the values, while the whiskers enclose 98% of the data. The median is indicated by a horizontal line and the mean represented by a small rectangle. (TIF) [file pone.0065837.s003.tif]

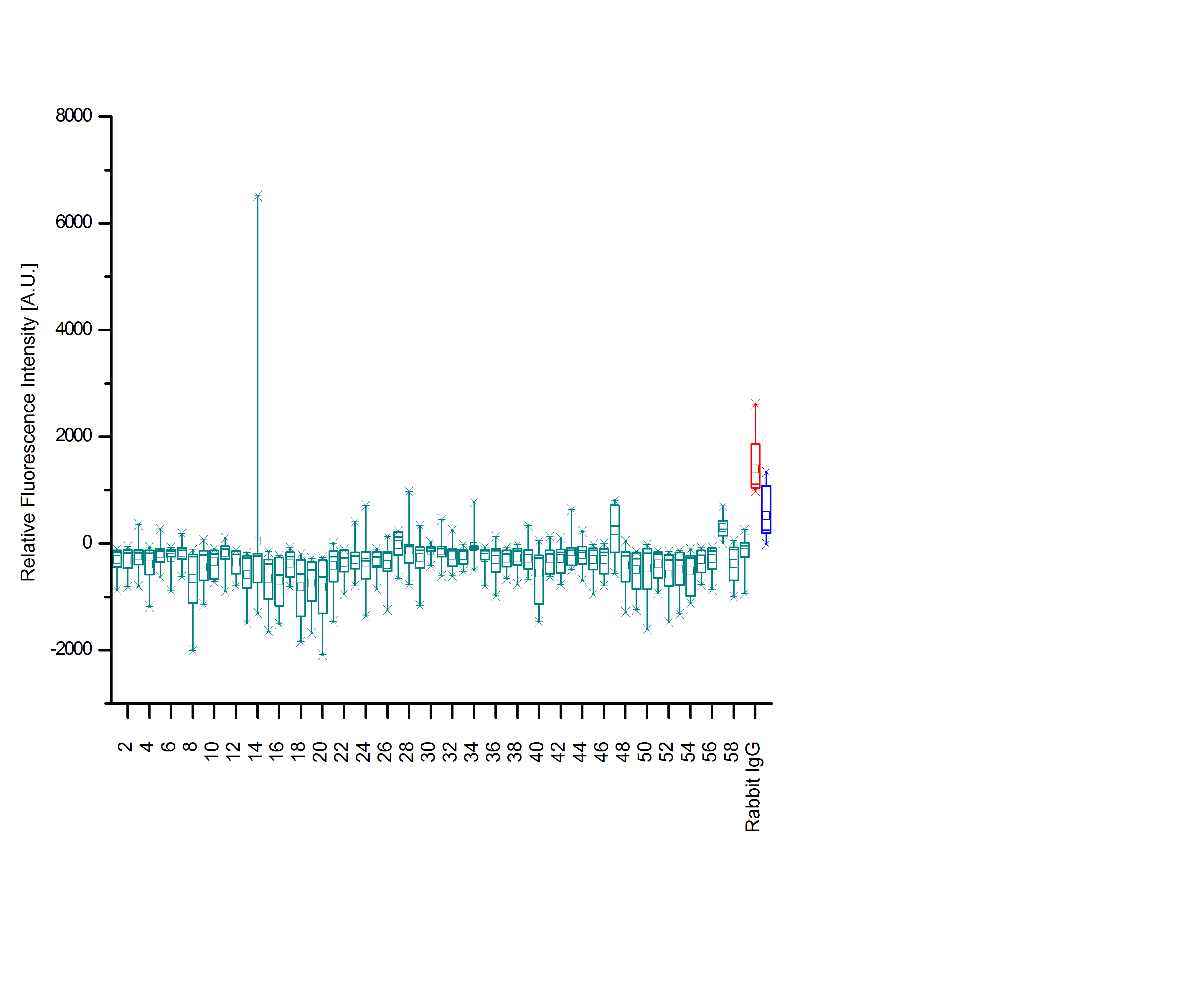

Supplement: Figure S4 — Epitope Mapping of cj0623. Box-whisker-plot (n = 15) showing the relative fluorescence intensities of the different overlapping peptides including Rabbit IgG (red) and MBP (blue) as positive and negative controls. Each Box represents 50% of the values, while the whiskers enclose 98% of the data. The median is indicated by a horizontal line and the mean represented by a small rectangle. (TIF) [file pone.0065837.s004.tif]

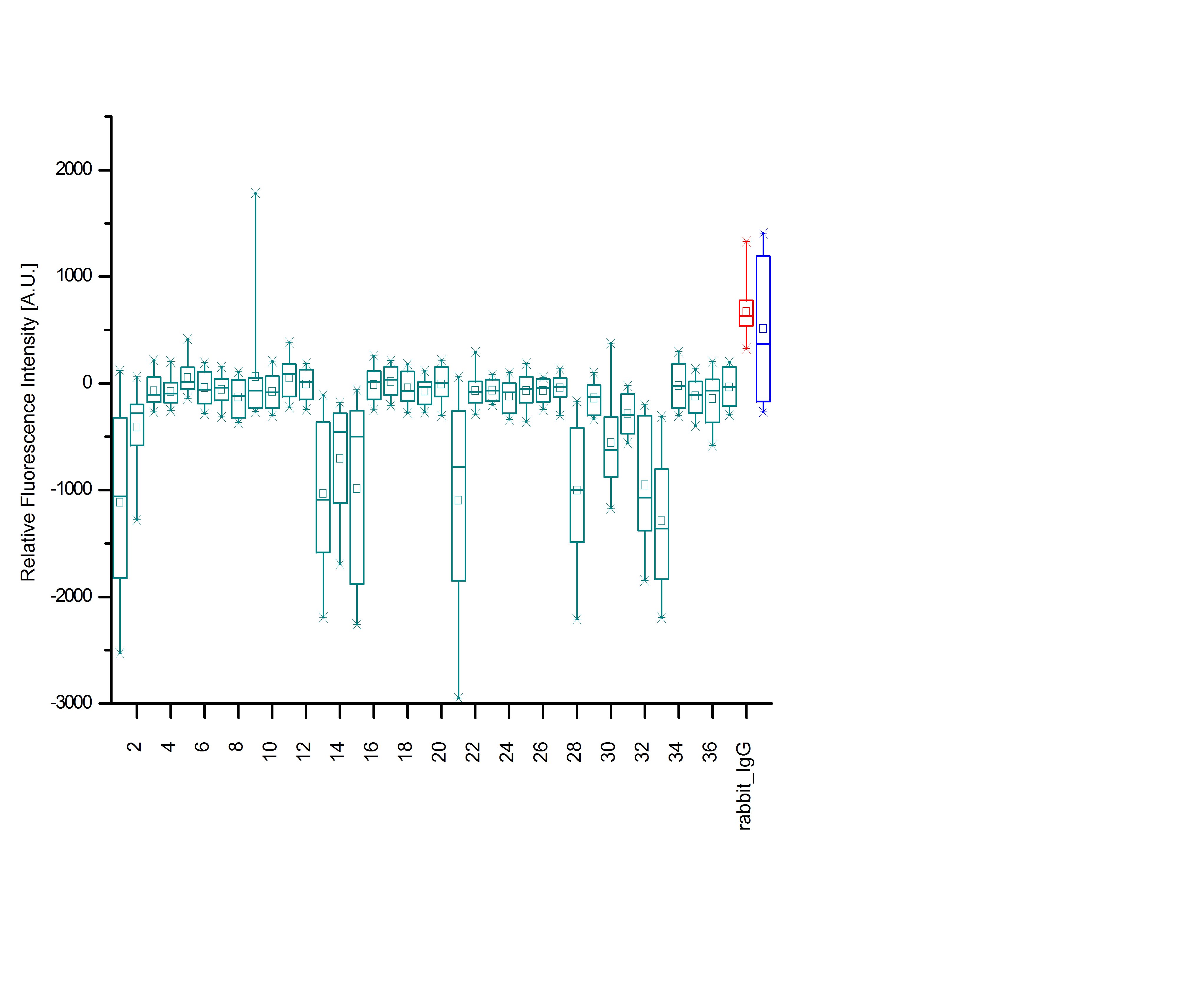

Supplement: Figure S5 — Epitope Mapping of cj0476. Box-whisker-plot (n = 12) showing the relative fluorescence intensities of the different overlapping peptides including Rabbit IgG (red) and MBP (blue) as positive and negative controls. Each Box represents 50% of the values, while the whiskers enclose 98% of the data. The median is indicated by a horizontal line and the mean represented by a small rectangle. (TIF) [file pone.0065837.s005.tif]

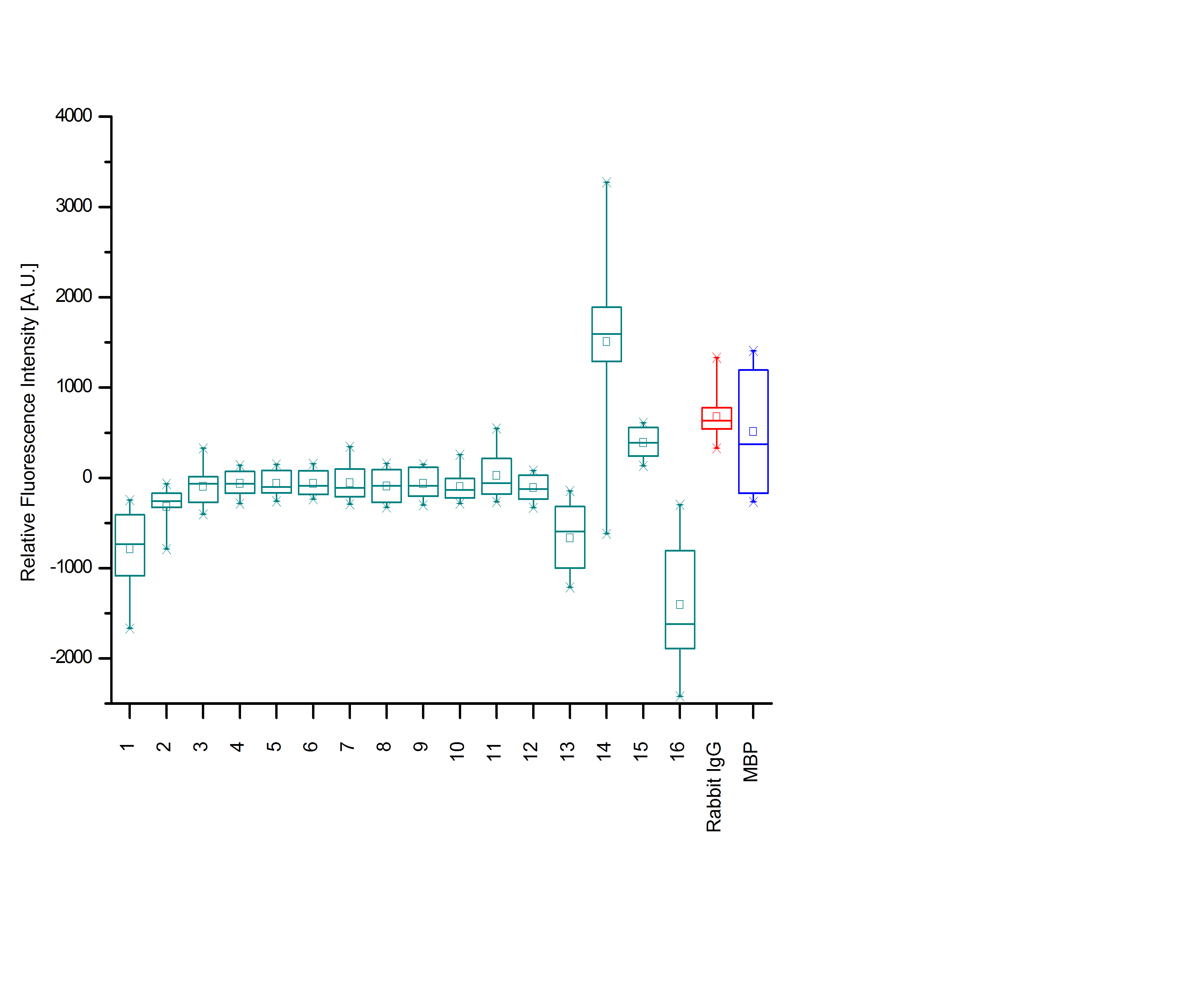

Supplement: Figure S6 — Epitope Mapping of cj1723. Box-whisker-plot (n = 12) showing the relative fluorescence intensities of the different overlapping peptides including Rabbit IgG (red) and MBP (blue) as positive and negative controls. Each Box represents 50% of the values, while the whiskers enclose 98% of the data. The median is indicated by a horizontal line and the mean represented by a small rectangle. (TIF) [file pone.0065837.s006.tif]

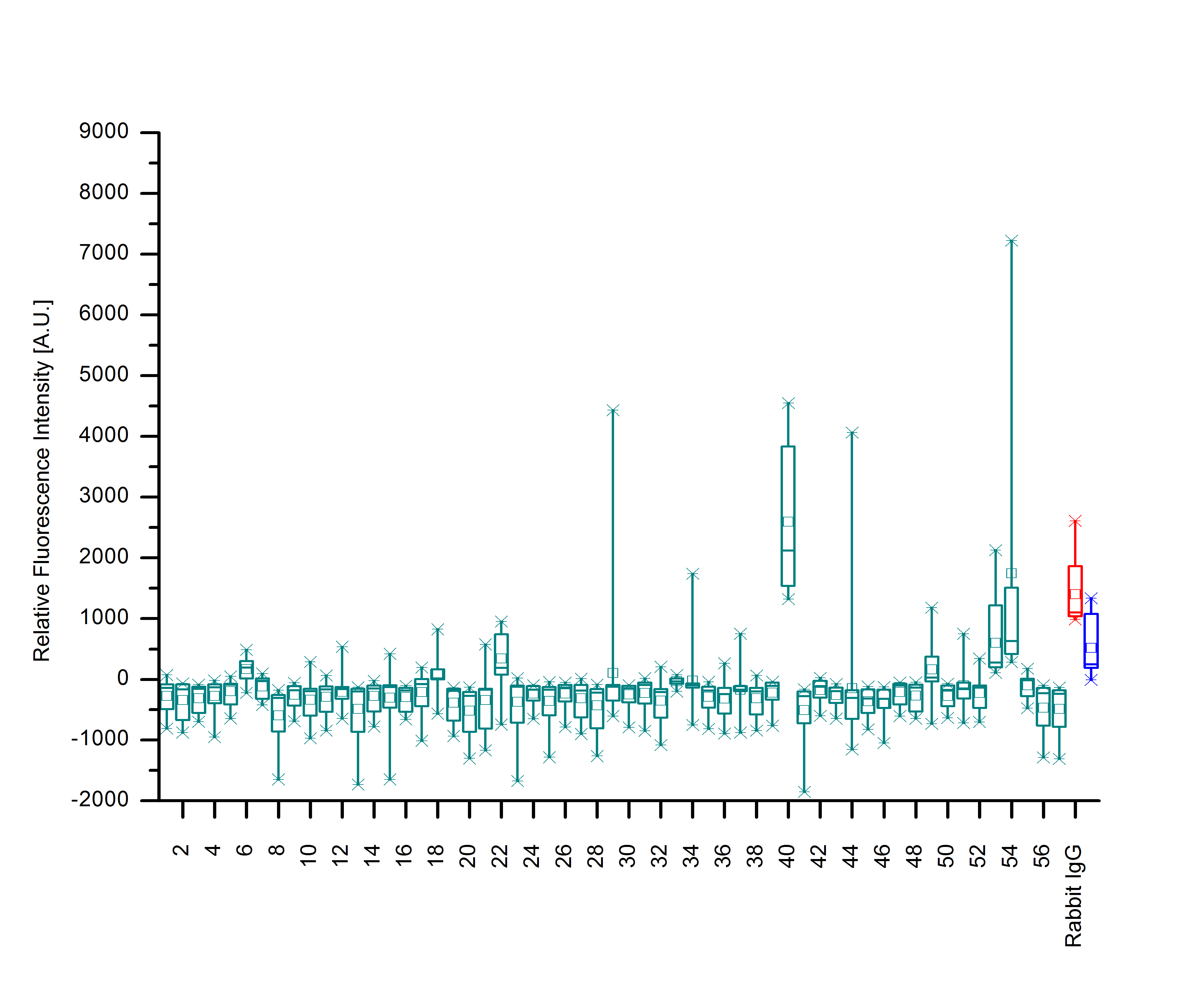

Supplement: Figure S7 — Epitope Mapping of cj1380. Box-whisker-plot (n = 15) showing the relative fluorescence intensities of the different overlapping peptides including Rabbit IgG (red) and MBP (blue) as positive and negative controls. Each Box represents 50% of the values, while the whiskers enclose 98% of the data. The median is indicated by a horizontal line and the mean represented by a small rectangle. (TIF) [file pone.0065837.s007.tif]

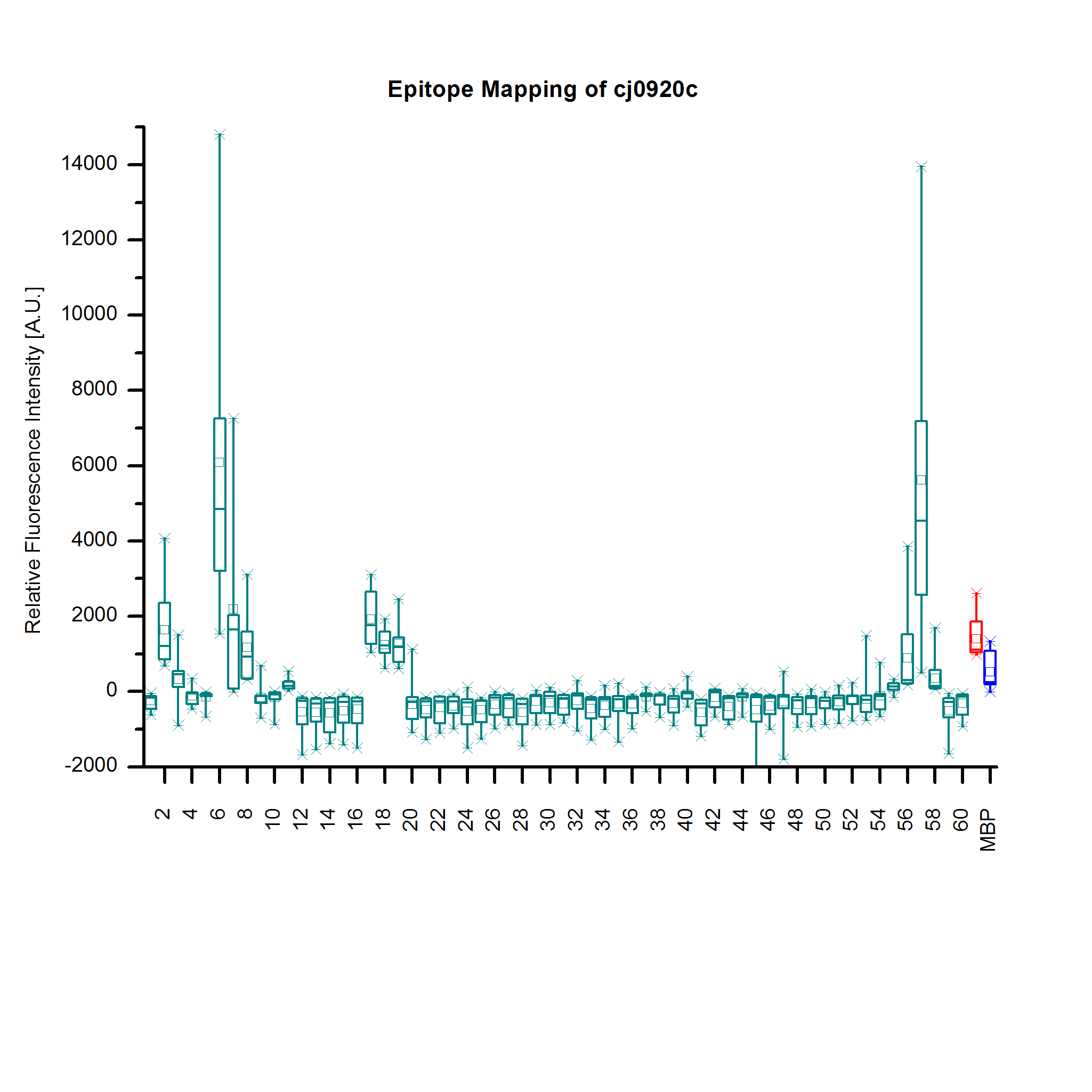

Supplement: Figure S8 — Epitope Mapping of cj0920c. Box-whisker-plot (n = 15) showing the relative fluorescence intensities of the different overlapping peptides including Rabbit IgG (red) and MBP (blue) as positive and negative controls. Each Box represents 50% of the values, while the whiskers enclose 98% of the data. The median is indicated by a horizontal line and the mean represented by a small rectangle. Several parts of the protein show intensities above the positive control, namely peptides 17 to 19, 56 and 57 as well as peptides 6 to 8. This indicates potential antigenic sites within the above peptides. (TIF) [file pone.0065837.s008.tif]

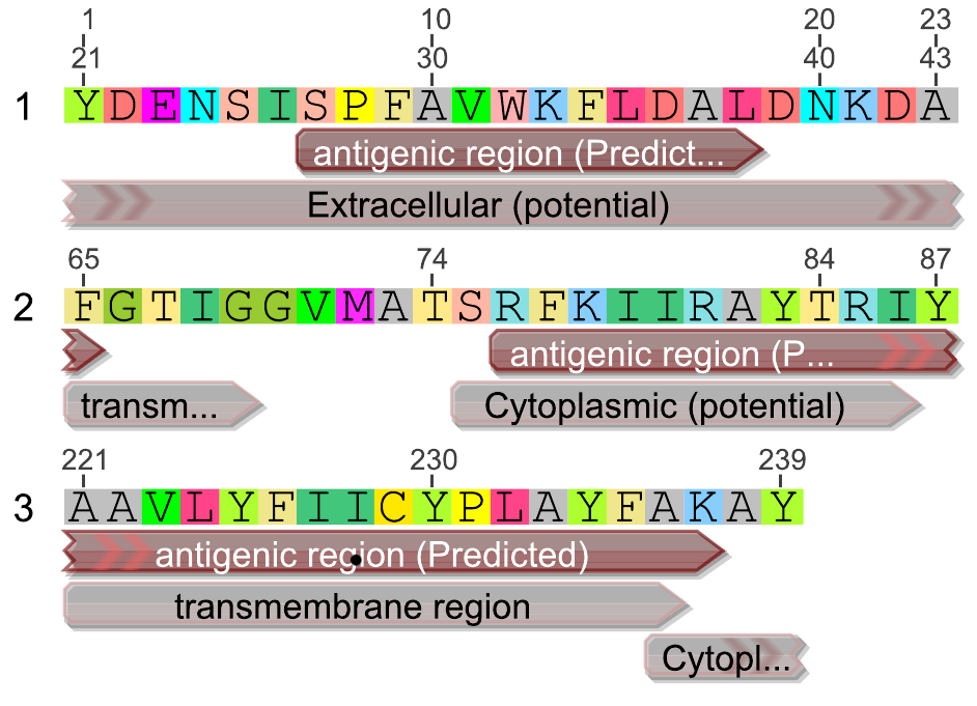

Supplement: Figure S9 — Transmembrane and antigenic potential of three potential epitope sites for cj0920c. Shown are the three regions represented by peptides 6–8 (aa 21–43), 17–19 (aa 65–87) and 56–57 (aa 221–239). Antigenic regions were predicted by EMBOSS antigenic with a minimum size of 5 residues. Transmembrane regions were predicted using TMHMM2.0. For the sequence SPFAVWKFLDAL both antigenic site and extracellular position are predicted, while the other amino acids are either not antigenic or located within the cytoplasm or transmembrane regions. (TIF) [file pone.0065837.s009.tif]

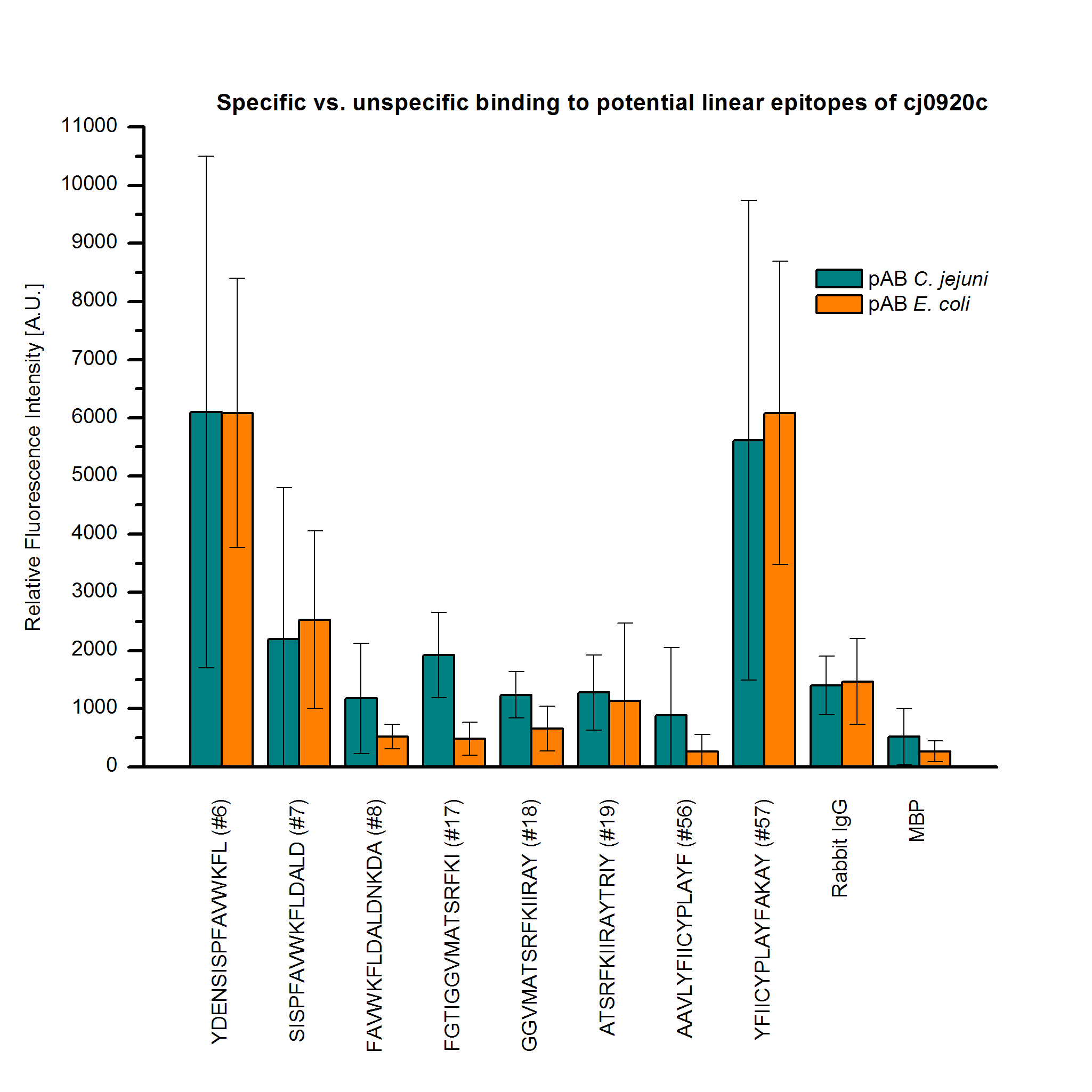

Supplement: Figure S10 — Specific vs. non-specific binding to potential linear epitopes of cj0920c. The bars represent the mean values (n = 15) of rfi values after incubation with polyclonal antibody to C. jejuni (green) and Salmonella enterica (orange). The mean values for each peptide fall within the same range or possess overlapping standard deviations. Thus, no specific interaction of the antibody to the epitope is present; rather a non-specific binding seems likely. (TIF) [file pone.0065837.s010.tif]

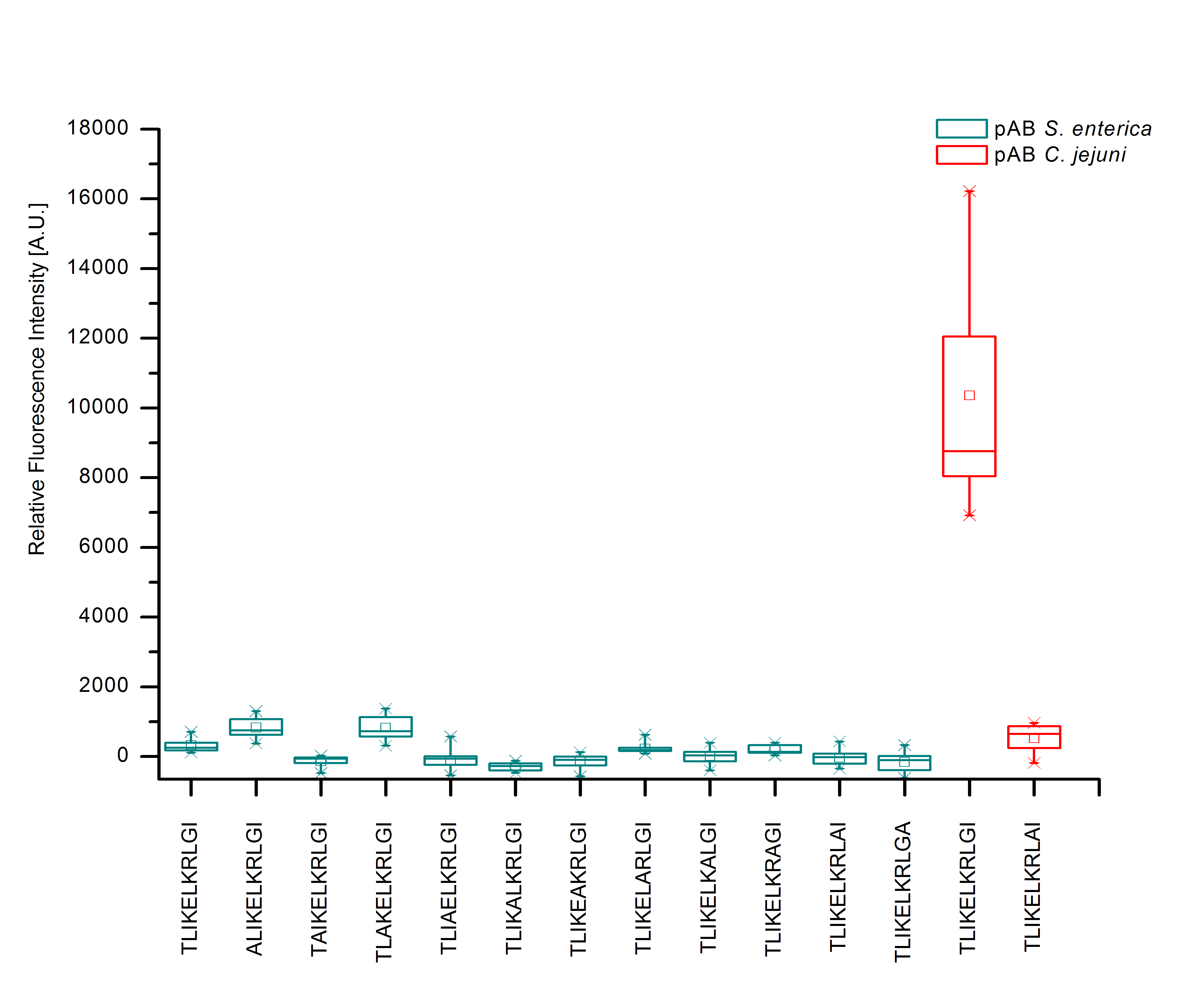

Supplement: Figure S11 — Binding specificity assay of TLIKELKRLGI with anti- Salmonella antibodies. The different sequences tested in alanine scanning are shown in the box-whisker-plot (n = 15) with each box representing 50% of the values. The whiskers encompass 98% of the values, the median is indicated by a horizontal line and the mean represented by a small rectangle. The 12 boxes in green on the left represent the results after incubation with polyclonal antibody to S. enterica. For comparison, the two red boxes show the original signals from Fig. 4 for the sequence TLIKELKRLGI as well as TLIKELKRLAI, after incubation with polyclonal antibodies to C. jejuni. All the green boxes fall into the same range as the altered sequence TLIKELKRLAI, where alanine replaced the glycine residue, which possessed only 10% intensity of the original sequence. Thus, no specific interaction of the antibody to the epitope is present; rather a non-specific binding seems likely. (TIF) [file pone.0065837.s011.tif]
