# Supplementary material for: Rapid Identification of Novel Immunodominant Proteins and Characterization of a Specific Linear Epitope of Campylobacter jejuni
Source: PLoS One. 2013 May 29;8(5):e65837. doi: 10.1371/journal.pone.0065837 (PMC3667084; doi:10.1371/journal.pone.0065837)
Supplement: Figure S2 — LIKELKRLGI is conserved in C. jejuni . The protein sequence of Cj0669 was BLASTed and subsequently aligned according to a BLOSUM62 matrix. As a reference sequence TLIKELKRLGI of Q0PAK5, the protein encoded by cj0669 from C. jejuni NCTC 11168 was used. The dots indicate an agreement to the reference, while differences are given by the one letter amino acid code. The first 13 sequences including the references are 100% identical and are all derived from C. jejuni proteins. For other species of Campylobacter such as C. coli or C. upsaliensis several of the residues are replaced. For other organisms, especially Helicobacter pylori the degree of replaced residues increases. The positions showing the most conservation throughout are residues 3, 6, 9 and 11. However, residue 10, the glycine which was revealed to be paramount for the binding of the antibody is not found in Helicobacter pylori proteins. (PDF) [file pone.0065837.s002.pdf]

	1 … 10 11
Consensus	XI.XX.XX.N.
Identity	
	177 … 187
1. Campylobacter jejuni	TLIKELKRLGI
2. Campylobacter jejuni subsp. jejuni CG8486	....
3. Campylobacter jejuni subsp. jejuni CG8421	....
4. Campylobacter jejuni RM1221	....
5. Campylobacter jejuni subsp. jejuni 260.94	....
6. Campylobacter jejuni subsp. jejuni LMG 23223	....
7. Campylobacter jejuni subsp. jejuni D2600	....
8. Campylobacter jejuni subsp. jejuni 414	....
9. Campylobacter jejuni subsp. jejuni CF93-6	....
10. Campylobacter jejuni subsp. jejuni 2008-979	....
11. Campylobacter jejuni subsp. jejuni 60004	....
12. Campylobacter jejuni	....
13. Campylobacter jejuni subsp. jejuni DFVF1099	....
14. Campylobacter coli 111-3	S..N...K.D.
15. Campylobacter coli RM2228	S..N...K.D.
16. Campylobacter coli 1098	S..N...K.D.
17. Campylobacter upsaliensis JV21	N........D.
18. Campylobacter upsaliensis RM3195	N........D.
19. Campylobacter lari RM2100	S...D..AMN.
20. Campylobacter fetus subsp. fetus 82-40	GI.ND..K.N.
21. Campylobacter showae RM3277	SIVRD..K...
22. Campylobacter rectus RM3267	SIVRD..K...
23. Campylobacter curvus 525.92	SIVRD..N...
24. Campylobacter concisus 13826	SIVRD..K...
25. Campylobacter sp. 10_1_50	SIVRD..K...
26. Campylobacter hominis ATCC BAA-381	NIV.S..D...
27. Sulfurospirillum barnesii SES-3	SIVQD.SK...
28. Sulfurospirillum deleyianum DSM 6946	SIVQ..SK...
29. Arcobacter nitrofigilis DSM 7299	EI.NQ.TLR..
30. Campylobacter gracilis RM3268	SIVRD.SD...
31. Arcobacter sp. L	EI.HQ.TKIN.
32. Arcobacter butzleri RM4018	EI.HQ.TKIN.
33. Helicobacter hepaticus ATCC 51449	NI.LR.TE.D.
34. Helicobacter cinaedi CCUG 18818	NI.LK.TQ...
35. Helicobacter cinaedi PAGU611	NI.LK.TQ...
36. uncultured Sulfuricurvum sp. RIFRC-1	SV.SQ.VEY..
37. Sulfurimonas denitrificans DSM 1251	.V..Q.VSY..
38. Sulfurovum sp. AR	NI..Q.VD.DM
39. Helicobacter bilis ATCC 43879	KI.QT.VEMD.
40. Helicobacter felis ATCC 49179	KI.EH.VEMN.
41. Helicobacter mustelae 12198	KI.QK.VK.D.
42. Sulfuricurvum kujiense DSM 16994	GV.SQ.VEY..
43. Helicobacter cetorum MIT 00-7128	KI.ES.IE.N.
44. Helicobacter pylori Aklavik117	KI.ES.IK.N.
45. Helicobacter cetorum MIT 99-5656	KI.ES.VE.N.
46. Nitratiruptor sp. SB155-2	KV.N...KS..
47. Helicobacter suis HS1	KI.EH.VERK.
48. Helicobacter pylori Shi417	KI.ES.IK.N.
49. Helicobacter pylori CPY1662	KI.ES.IE.N.
50. Sulfurimonas gotlandica GD1	KV.HQ.VSYD.
51. Helicobacter pylori 83	KI.ES.IK.N.
52. Helicobacter bizzozeronii CIII-1	KI.ER.VEMN.
53. Helicobacter pylori FD423	KI.QS.IE.N.
54. Helicobacter pylori CPY3281	KI.EN.IG.N.
55. Helicobacter pylori CPY1124	KI.ES.IG.N.
56. Helicobacter pylori PeCan4	KI.ES.IG.N.
57. Helicobacter pylori Shi470	RI.ES.IG.N.
58. Helicobacter pylori Hp P-41	RI.ES.IG.N.
59. Helicobacter pylori Aklavik86	KI.ES.IG.N.
60. Helicobacter pylori F32	KI.ES.IK.N.
61. Helicobacter pylori GAM264Ai	RI.ES.IG.N.
62. Helicobacter pylori GAM114Ai	RI.ES.IG.N.
63. Helicobacter pylori HP116Bi	RI.ES.IG.N.
64. Helicobacter pylori SouthAfrica7	KI.ES.IE.N.
65. Helicobacter pylori Hp A-6	RI.ES.IG.N.
66. Helicobacter pylori Hp H-36	RI.ES.VG.N.
67. Helicobacter pylori 98-10	KI.ES.IG.N.
68. Helicobacter pylori GAM260BSi	KI.ES.IG.N.
69. Helicobacter pylori NQ4044	KI.ES.IE.N.
70. Helicobacter pylori Hp H-28	KI.ES.IE.N.
71. Helicobacter pylori J99	RI.ES.IG.N.
72. Helicobacter pylori PeCan18	KI.ES.IG.N.
73. Helicobacter pylori GAM71Ai	RI.ES.IG.N.
74. Helicobacter pylori NQ392	KI.ES.IG.N.
75. Helicobacter acinonychis str. Sheeba	KI.ES.IE.N.
76. Helicobacter pylori 2018	KI.ES.IG.N.
77. Wolinella succinogenes DSM 1740	RI..Q.VDF..
78. Helicobacter pylori Hp A-4	RI.ES.IG.N.
79. Helicobacter pylori Hp A-8	KI.ES.IG.N.
80. Helicobacter pylori SNT49	KI.QS.IG.N.
81. Helicobacter pylori GAM201Ai	RI.ES.IG.N.
82. Helicobacter pylori R030b	KI.ES.IG.N.
83. Sulfurimonas autotrophica DSM 16294	KV.HQ.ISYD.
84. Helicobacter pylori 26695	KI.ES.IG.N.
85. Helicobacter pylori B128	KI.ES.IG.N.
86. Helicobacter pylori Hp A-17	RI.ES.IG.N.
87. Helicobacter pylori Gambia94/24	RI.ES.IG.N.
88. Helicobacter pylori Hp P-3	KI.ES.IG.N.
89. Helicobacter pylori India7	RI.ES.IG.N.
90. Helicobacter pylori HUP-B14	KI.ES.IG.N.
91. Nitratifractor salsuginis DSM 16511	AI.RQ.RD.D.
92. Helicobacter pylori HPKX_438_AG0C1	KI.ES.IG.N.
93. Helicobacter pullorum MIT 98-5489	NI..K.LKFN.
94. Helicobacter pylori Hp H-41	KI.ES.IG.N.
95. Helicobacter pylori NQ4053	KI.ES.IG.N.
96. Sulfurovum sp. NBC37-1	NI.RQ.VE.DM
97. Caminibacter mediatlanticus TB-2	D...L.SNQN.
98. Nautilia profundicola AmH	H.VEL.AKEN.
99. Helicobacter canadensis MIT 98-5491	NI..K.LKF..
100. Thiovulum sp. ES	QV..Q..EMN.
101. Helicobacter pylori Hp P-13	KI.ES.IG.N.
